# The Plastid-Localized AtFtsHi3 Pseudo-Protease of *Arabidopsis thaliana* Has an Impact on Plant Growth and Drought Tolerance

**DOI:** 10.3389/fpls.2021.694727

**Published:** 2021-06-23

**Authors:** Laxmi S. Mishra, Sanatkumar Mishra, Daniel F. Caddell, Devin Coleman-Derr, Christiane Funk

**Affiliations:** ^1^Department of Chemistry, Umeå University, Umeå, Sweden; ^2^Umeå Plant Science Center, Department of Forest Genetics and Plant Physiology, Swedish University of Agricultural Sciences, Umeå, Sweden; ^3^Plant Gene Expression Center, US Department of Agriculture-Agricultural Research Service, Albany, CA, United States; ^4^Department of Plant and Microbial Biology, University of California, Berkeley, Berkeley, CA, United States

**Keywords:** drought, filamentation temperature-sensitive H protease, root microbiome, abscisic acid, *Arabidopis thaliana*, chloroplast

## Abstract

While drought severely affects plant growth and crop production, the molecular mechanisms of the drought response of plants remain unclear. In this study, we demonstrated for the first time the effect of the pseudo-protease AtFtsHi3 of *Arabidopsis thaliana* on overall plant growth and in drought tolerance. An *AtFTSHi3* knock-down mutant [*ftshi3-1(kd)*] displayed a pale-green phenotype with lower photosynthetic efficiency and Darwinian fitness compared to wild type (Wt). An observed delay in seed germination of *ftshi3-1(kd)* was attributed to overaccumulation of abscisic acid (ABA); *ftshi3-1(kd)* seedlings showed partial sensitivity to exogenous ABA. Being exposed to similar severity of soil drying, *ftshi3-1(kd)* was drought-tolerant up to 20 days after the last irrigation, while wild type plants wilted after 12 days. Leaves of *ftshi3-1(kd)* contained reduced stomata size, density, and a smaller stomatic aperture. During drought stress, *ftshi3-1(kd)* showed lowered stomatal conductance, increased intrinsic water-use efficiency (WUEi), and slower stress acclimation. Expression levels of ABA-responsive genes were higher in leaves of *ftshi3-1(kd)* than Wt; *DREB1A*, but not *DREB2A*, was significantly upregulated during drought. However, although *ftshi3-1(kd)* displayed a drought-tolerant phenotype in aboveground tissue, the root-associated bacterial community responded to drought.

## Introduction

Drought is an environmental stress that can negatively impact plant productivity and crop yields (Gupta et al., 2020). Several classes of proteases are involved in the acclimation of plants to drought (reviewed by Vaseva et al., 2011; Fanourakis et al., 2020). For example, the senescence-associated subtilisin protease (SASP) is a key regulator in abscisic acid (ABA) signaling and drought tolerance (Acharya et al., 2013; Wang et al., 2018b). Cysteine proteinases belonging to the vacuolar processing enzymes (VPEs; reviewed by Hatsugai et al., 2015; Vorster et al., 2019) participate in controlling stomatal pore aperture during both pathogen attack and drought stress (Albertini et al., 2014; Lu et al., 2016). Overexpression of the S8 subtilisin-like serine protease (Berger and Altmann, 2000) correlates with decreased stomatal density, enhanced water-use efficiency (WUEi), and drought tolerance (Yoo et al., 2010; Liu et al., 2015; Morales-Navarro et al., 2018). The molecular mechanisms underpinning protease involvement in responses to water deficit remain unclear.

The membrane-embedded filamentation temperature-sensitive H (FtsH) protease family members play an essential role in the degradation of both soluble and membrane proteins and are extensively studied in bacteria, chloroplasts, and mitochondria (Smakowska et al., 2014; Nishimura et al., 2016; Bittner et al., 2017). Annotated members of the FtsH family contain a putative AAA ATPase domain (IPR003593), an ATPase AAA core (IPR003959), and an M41 peptidase domain (IPR000642; https://Arabidopsis.org). In addition to the proteolytically active FtsH proteases, pseudo-proteases, termed FtsHi (i for inactive Sokolenko et al., 2002), have been detected in the genomes of plants. *Arabidopsis thaliana* encodes five FtsHi enzymes, of which the consensus sequence HEXXH present in the Zn^+^-binding M41 metalloprotease domain is either altered (FtsHi1/2/4/5) or absent (FtsHi3; Wagner et al., 2012). Homozygous mutants deficient in FtsHi1, 2, 4, or 5 are seed lethal (Kadirjan-Kalbach et al., 2012; Lu et al., 2014; Wang et al., 2018c; Mishra et al., 2019). “Weak” point mutations or heterozygous *FTSHi* mutants display a pale-seedling phenotype, the plants have smaller rosette sizes throughout their life span (Kadirjan-Kalbach et al., 2012; Lu et al., 2014) and often contain variegated leaves (Wang et al., 2018c). Homozygous *ftshi3* is not embryo lethal, but knockdown plants containing about 10% FtsHi3 of wild type (Wt) level display a pale seedling phenotype (Kikuchi et al., 2018; Mishra et al., 2019). Seeds from field-grown *ftshi3-2* plants germinated with a significant delay compared to the Wt control (Mishra et al., 2019).

The chloroplast envelope-located FtsHi1, 2, 4, and 5 were found to form a complex with FtsH12 and NAD-dependent malate dehydrogenase (pdNAD-MDH), which functions as a motor for protein translocation into the chloroplast (Kikuchi et al., 2018; Schreier et al., 2018). Even Ycf2 might be part of this complex (Kikuchi et al., 2018). The fifths pseudo-protease, FtsHi3, was instead detected in a separate 1-MD complex (Kikuchi et al., 2018); its function and the identity of the other complex components remain unclear.

In this study, we describe an *Arabidopsis ftshi3* mutant that shows a drought-tolerant phenotype above ground but accumulates drought-specific root-associated bacterial communities below ground. Unlike the previously investigated *FTSHi3* knockdown lines *ftshi3-2* (GK_723C06) and *ftshi3* with residual *FTSHi3* expression (Kikuchi et al., 2018; Mishra et al., 2019), the knock-down mutant *ftshi3-1(kd*; GK-555D09-021662) had fully reduced transcription of *FTSHi3* and displayed a pale-green phenotype throughout its life span. Characterizing this mutant in the field and controlled conditions, we were able to show that the absence of FtsHi3 impacts leaf stomatal density, lowered stomatal conductance, and increased intrinsic WUEi. The mutant expressed higher levels of the *DREB1A* in watered and drought conditions, but *DREB2A* only in watered conditions. Therefore, its drought-tolerant phenotype was attributed to an ABA-independent pathway.

## Materials and Methods

### Plant Material and Growth Conditions

*Arabidopsis thaliana* ecotype *Columbia-0* (wild type, Wt) and *ftshi3-1* (GabiKat-555D09-021662; Kleinboelting et al., 2012) transfer DNA (T-DNA) mutant seeds were obtained from the Nottingham Arabidopsis Stock Center (NASC). T-DNA insertion was confirmed by PCR and sequencing-based methods. Primers used for genotyping and sequencing are listed in Supplementary Table S1.

*Arabidopsis* Wt and mutant seeds were sterilized with 10% NaOCl, washed four times with sterile water, and then stratified for 2 days at 4°C. The seeds were selected on full-strength MS agar (Murashige and Skoog, 1962), supplemented with 1% sucrose and 75 μg/L sulfadiazine. After growing on plates for 12 days post-germination, the plants were transferred to soil. Stress treatments with mannitol or ABA were performed on agar plates 7 days post-germination. Seedlings were moved with sterile forceps, allowed to grow in the presence of 200 mM mannitol with 1 or 5 μM ABA for 7 days, and collected to determine their dry weight. Other stress experiments were performed as described by Mishra et al. (2019).

### Genotyping and Whole-Genome Sequencing

To confirm the *ftshi3-1(kd)* mutation, high-quality genomic DNA was extracted from the T-DNA insertion lines (Healey et al., 2014; O’malley et al., 2015) and submitted to the BGI European Genome Center for whole-genome sequencing (WGS) Plant DNA sequencing. Next generation sequencing (NGS) data pre-processing was performed at the Umeå Plant Science bioinformatics facility, Sweden, following a standard pipeline.

The sequencing was performed on an Illumina HiSeq4000 sequencer for 150 cycles in paired-end mode. The quality of the reads was assessed using FastQC^1^, v0.11.4. The data were deemed excellent, and neither no quality-based trimming nor adapter removal was necessary. The TAIR 10 version of the *Arabidopsis* genome was retrieved from the TAIR resource (Berardini et al., 2015), and the sequence of the tDNA pAC161 plasmid was added to that reference. BWA version 0.7.17 (Li, 2013) was used to index the extended genome reference and align the paired-end reads. Adding the T-DNA plastid sequence allowed for confirming its insertion position and uniqueness. Subsequently, GATK version 4.0.8.1 (Van Der Auwera et al., 2013) was used to process the data following the guidelines of GATK, namely applying the following tools in series: BaseRecalibrator, ApplyBQSR, BaseRecalibrator, AnalyzeCovariates, and HaplotypeCaller. All tools were used with their default settings apart from enabling parallel processing whenever possible. Downstream analyses were conducted in (R Core Team, 2020), version 3.5.1 using Bioconductor (Huber et al., 2015) core and the VariantAnnotation (Obenchain et al., 2014) packages. All scripts are available from the GitHub repository: https://github.com/LSmishra/FtsHi3. The data are available from the European Nucleotide Archive, www.ebi.ac.uk/ena, under the accession ID: PRJNA669866.

### Plasmid Construction and Plant Transformation

To construct complementation and knockdown lines, genomic DNA fragments were amplified from *A. thaliana* by Phusion ®(Thermo Scientific, United States) proofreading polymerase. Knockdown *FTSHi3* lines [microRNA (*miRNA)-a* and *miRNA-b*] were generated *via* micro-RNA constructs according to WMD3 – Web MicroRNA Designer (http://wmd3.weigelworld.org/cgi-bin/webapp.cgi; Schwab et al., 2006), with specific primers designed for ‘*miRNA-a* and *miRNA-b* (I/II/III/IV miR–s/−a/*s/*a’; Supplementary Table S1), targeting two independent regions of *FTSHi3* (AT3G02450). PCR fragments were cloned into the gateway-compatible plasmid RS300 (MIR319a). The construct was recombined into the estrogen-inducible vector pMDC7 for silencing *FTSHi3*. Wt plants were transformed with these constructs, and T1 seedlings were selected based on their sensitivity to β-estradiol and hygromycin-B resistance. Young seedlings were selected by plating them on MS agar plates containing β-estradiol, whereas, in older plants, the entire rosettes were sprayed with a β-estradiol solution every second day. T2 plants were used for further analysis. A construct containing the amplified *FTSHi3* promoter sequence (predicted according to Knudsen, 1999) was generated using the primers “ftshi3 Promoter Forward” and “ftshi3 Reverse for HA-line” (Supplementary Table S1). The *pAtftshi3::ftshi3* genomic DNA was cloned into a pENTR/D-TOPO vector and transferred into the destination vector, pGWB15, resulting in a 3xHA tagged gene product. The binary plasmids were transformed into electro-competent *Agrobacterium tumefaciens* [GV3101::pMP90 (pTiC58DT-DNA); Hellens et al., 2000]. The non-segregating T2 homozygous *ftshi3-1* T-DNA line of the GABI-KAT collection was transformed as described by Clough and Bent (1998). The construct in the T1 and T2 generation was confirmed by germinating transgenic seeds on 35 mg/ml hygromycin-B selecting MS agar plates. The experiments were performed on T3 generation seeds.

### Field Experiments and Darwinian Fitness Analysis

Field experiments, including Darwinian fitness analysis, were performed in Umeå, Sweden (63°49'07.2"N 20°18'45.0"E) during the weeks 26–36 in 2017 as described in Frenkel et al. (2008). After germinating the seeds on plates, the seedlings were grown at long-day conditions (LD, 21°C, 16-h light/8-h dark) in the greenhouse for 12–14 days. The plants were moved in the field, acclimatized for 24 h, and then grown in semi-natural conditions in the field. After 11 weeks in the field, the plants were moved indoors to dry their seeds and siliques for Darwinian fitness analysis (Mishra et al., 2019). SPSS software was used to perform statistical analyses. The values of *p* were calculated with an ANOVA and a least significant difference (LSD) *post hoc* test and Student’s *t* test. Weather data were obtained from TFE Väder – info (umu.se) maintained by the Department of Applied Physics and Technology, Umeå University, Sweden. The data were collected at a station 650 m from the actual field site (Mishra et al., 2019).

### Drought Phenotype Analysis

Water-deficit stress was applied on plants grown on the soil at a relative humidity of 50% and 150 μmol photons m^−2^ s^−1^ for approximately 4 weeks before treatment. Plants were exposed to short-day (SD) conditions (8/16 h photoperiod, 22/18°C) or LD conditions (16/8 h photoperiod, 22/18°C).

Severe drought treatment was induced by stopping the irrigation when plants were 4 weeks old until the genotypes showed drought effects compared to the well-watered controls. These controls were watered daily with 550 ml water per tray (15 pots/tray). The pot weight of samples and controls was determined by weighing plants and soil. After 20 days of severe drought, all genotypes were re-watered daily with 350 ml of water for 14 days, and the number of plants resuming their growth was counted. These experiments were performed three times with 10 biological replicates each.

### Plant Growth Conditions During the Microbiome Experiment

Wild type, the T-DNA insertion line *ftshi3-1* (Gabi Kat-555D09-021662), and lines expressing the *pAtFtsHi3::AtFtsHi3::HA* (complementation lines) were used to study the microbiome during watered and drought conditions. Surface sterilized seeds were plated on half MS agar media, vernalized at 4°C for 2–3 days, and then moved to a growth chamber exposing them to SD conditions (8/16 h photoperiod, 22/18°C and 120 μmol photons m^−2^ s^−1^) with a relative humidity of 70% for approximately 2 weeks. The 2-week-old seedlings were transferred to 5 cm square plastic plant pots with a soil mixture containing 10% field soil, 60% sunshine potting mix, and 30% vermiculite. Field soil had been collected from an agricultural field site located in Albany, California, United States (37.8864°N, 122.2982°W, Simmons et al., 2018). Plants were randomized by treatment, genotype, and replication, with five replicates per genotype and treatment. The plants were allowed to grow at SD conditions (8/16 h photoperiod, 22/18°C and 120 μmol photons m^−2^ s^−1^) and were regularly watered with 750 ml of water per tray (each containing 15 randomized pots). At a plant age of 4 weeks, the watered control trays received 850 ml of water, while plants exposed to drought treatment received 450 ml of water per tray for 2–3 weeks. Then, the roots were harvested for genomic DNA extraction and 16S sequencing. Microbiome sample collection and amplicon sequence data processing were performed as described by Mishra et al. (2020). Library preparation and amplicon sequence data processing were performed according to Simmons et al. (2018, 2020) and Xu et al. (2018). All raw reads are deposited in the NCBI Short Read Archive at accession PRJNA669866.

### Microbiome Statistical Analyses

All 16S statistical analyses were performed in R v3.6.1 (R Core Team, 2013). Scripts and datasets can be found at https://github.com/colemanderr-lab/Mishra-2021. To account for differences in sequencing read depth across samples, the data per sample were normalized by dividing its reads per amplicon sequence variant (ASV) by the sum of usable reads, resulting in a table of relative abundance frequencies. All sample data were normalized to perform alpha diversity calculations to an even read depth of 17,716 ASVs per sample. The Shannon index was determined with the estimate_richness function in phyloseq v1.30.0 (Mcmurdie and Holmes, 2013), and significance was tested by ANOVA using the aov function in the R stats package. Canonical Analysis of Principal coordinates (CAP) of Bray-Curtis distance was performed using the ordinate function in phyloseq v1.30.0 (Mcmurdie and Holmes, 2013), and pairwise PERMANOVA determined sample type separation with 10,000 permutations using the adonis and calc_pairwise_permanovas functions in vegan v2.5.6 (Dixon, 2003) and mctoolsr v0.1.1.2 (Carini et al., 2016). Indicator species analysis (Dufrêne and Legendre, 1997) was performed after removing low abundance ASVs from the dataset (less than 25 reads and presence in less than 5% of samples) using the indval function in labdsv v2.0-1 (Roberts, 2007). Significant indicators were identified as ASVs with *p* < 0.05 and indcls > 0.05 following 10,000 permutations.

### Phenotypic Characterization

Seedlings of Wt and the *ftshi3-1* T-DNA insertion mutant were studied using a Leica MZ9.5.

### Measurement of Chlorophyll and Chlorophyll Fluorescence Parameters

Chlorophyll *a* and *b* were extracted from 100 mg leaf material according to Porra et al. (1989).

Chlorophyll fluorescence was measured using a PAM-210 (Walz, Germany) on plants growing either in standard or stressed conditions. The measurements were performed on the seventh or eighth leaf per plant per genotype. A 1 s light saturation pulse of 3,000 μmol m^−2^ s^−1^ was used to record F0. About 8–10 independent biological replicates of 6-week-old whole plants were dark-adapted for 30 min, the maximum PSII quantum yield *Fv/Fm* was documented. The total measuring time was 120 s, with saturation pulses (width = 800 ms); data were collected every 10 s. The non-photochemical quenching (NPQ) capacity was determined, and a light intensity of increasing photosynthetically active radiation (PAR) up to a maximum of 2,000 μmol photons m^−2^ s^−1^ was applied for 20 min.

### Transmission Electron Microscopy

Transmission electron microscopy (TEM) was used to study the chloroplast morphology of the first true leaf of 12-day-old seedlings of Wt, *ftshi3-1(kd)*, and *ftshi3-1* (*Comp-1* and *Comp-2*). Sample preparation and microscopy were performed at the KBC electron microscopy platform.

### Abscisic Acid Extraction and Quantification

Sample preparation and extraction for Solid Phase Extraction (SPE) and ultra-high performance liquid chromatography mass spectrometry (UHPLC-MS/MS) were performed as described by Haas et al. (2021).

### RNA Extraction, Complementary DNA Synthesis, and Quantitative PCR

RNA extraction and complementary DNA (cDNA) synthesis were performed using Invitrogen™ RNAqueous Total RNA Isolation Kit. Isolated RNA was reverse-transcribed into cDNA using Thermo Scientific RevertAid First Strand cDNA Synthesis Kit. Real-Time Quantitative Reverse Transcription PCR (Quantitative RT-PCR) was performed using a Bio-Rad CFX96 thermocycler. The housing-keeping genes ubiquitin, tubulin, and actin (Czechowski et al., 2005) and gene-specific quantitative PCR (qPCR) primers are listed in Supplementary Table S1. The data were analyzed using the Bio-Rad CFX Manager 3.1 software. RNA was isolated from different tissues of 4-week-old plants (i.e., young flowers, leaves, buds, siliques, and stems) and roots of 2-week-old plants germinated on MS agar plates.

### Measurements of Stomatal Density and Aperture in Response to ABA Treatment

Four-week-old rosette leaves were excised and peeled by the scotch tape method described by Lawrence II et al. (2018) and floated in ABA (10 μM) dissolved in DMSO for 1.5 h, while the control was floated in a buffer containing only 0.1% (v/v) DMSO. Microscopic images of stomata were taken on a Leica DMi8 at X40 magnification. For stomata density, five microscope fields per leaf were evaluated with five replicates per genotype. Stomatal aperture was measured using ImageJ software. Significance (value of *p* < 0.05, Student’s *t* test) of the 45 stomatal aperture measurements was calculated using SPSS software.

### Leaf-Level Gas Exchange

A portable photosynthesis system (Li-6400xt, Li-Cor, Lincoln, NE, United States) was used to determine the photosynthesis rate (assimilation *AN*) and stomatal conductance (g_s_) as described by Tomeo and Rosenthal (2018). Assimilation *AN* was measured at a CO_2_ concentration (Cr) of 400μmol mol^−1^ air and PAR of 1,000μmol photons m^−2^s^−1^ on the seventh or eighth leaf of the rosette across six biological replicates per treatment per genotype. The leaf chamber temperature was standardized to 25°C, and the airflow to 250 μmols^−1^. Gas exchange parameters were determined during the daytime between 11 am and 4 pm. The ratio of *AN*/g_s_ calculates the WUEi.

## Results

### Impact of FtsHi3 on Plant Development

**Figure 1:**
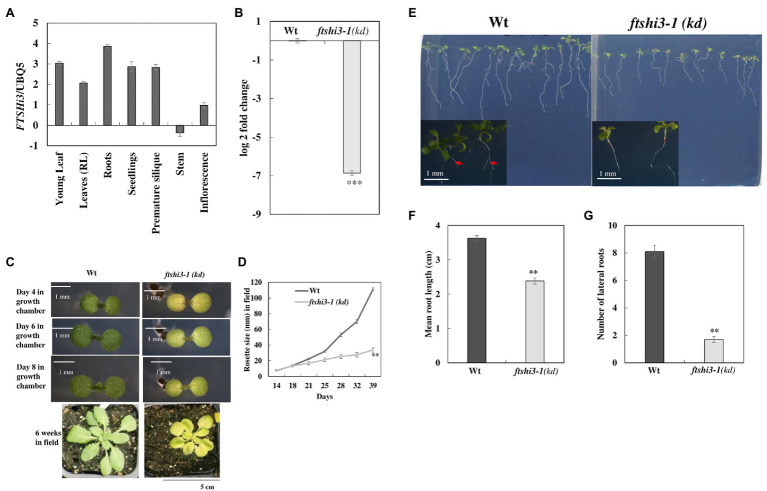
**(A)** Graphical representation of the organ-specific relative expression of *AtFTSHi3* (dCT values normalized to *At-UBQ*). **(B)** Log 2-fold change of *AtFTSHi3* transcript abundance in 4-week-old rosette leaves of Wt and *ftshi3-1(kd)*. The data are normalized to the expression of genes coding for *At-UBQ* and *At-Tubulin*. An asterisk indicates significant differences between Wt and the *ftshi3-1 (kd)* seedlings (**p < 0.05, Student’s *t* test, *n* = 3); error bars represent the SE. **(C)** Phenotypes of 4-, 6-, and 8-day-old seedlings of Wt and *ftshi3-1(kd)* grown in a growth chamber under short-day (SD) at 22°C (scale bars are 1 mm), or for 6 weeks under field conditions (Scale bar 5 cm). **(D)** Rosette diameter measurements were performed from 2 to 6 weeks on field-grown plants. Asterisks indicate a significant difference (**p < 0.05, Student’s *t* test, *n* = 8). **(E)** Root phenotype of 8-day-old seedlings from Wt and *ftshi3-1(kd)*. Arrows indicate the lateral roots. **(F,G)** Graphical representation of the root length **(F)** and the number of lateral roots (G; **p < 0.05, Student’s *t* test, *n* > 20).

To examine the role of AtFtsHi3 during growth and development, transcript levels of *AtFTSHi3* were analyzed in different tissues of Wt *Arabidopsis* plants (Figure 1A). AtFtsHi3 was ubiquitously expressed in all analyzed tissues, i.e., young flowers, roots, leaves, buds, siliques, and stems. T-DNA mutant seeds were obtained from the NASC. Expression of *FTSHi3* in the homozygous mutant *ftshi3-1* (GK-555D09-021662) [hereafter *ftshi3-1(kd)*] was fully reduced (99.18% reduction; Figure 1B), while 10% residual amounts of *FTSHi3* expression had been observed in *ftshi3-2* (Mishra et al., 2019). Analyzing the Darwinian fitness of various *FTSHi* mutants, the homozygous *ftshi3-2* mutant had displayed a pale-seedling phenotype, but in older plants, its phenotype did not differ from Wt (Mishra et al., 2019). The *ftshi3-2* phenotype was similar to *ftshi3* (FLAG_215F10; Kikuchi et al., 2018). Interestingly, the phenotype of *ftshi3-1* (GK-555D09-021662) differed from *ftshi3-2* (GK-723C06_025364) and *ftshi3* (FLAG_215F10; a schematic diagram of the location of the various T-DNA insertions is shown in Supplementary Figure S1A). The mutant displayed not only a pale-seedling phenotype but also remained pale throughout its life span (Figure 1C); it further grew significantly smaller than Wt (Figures 1C,D). We observed significantly reduced root growth (around 30%; Figures 1E,F) and lower numbers of lateral roots in *ftshi3-1(kd)* compared to Wt (Figure 1G).

### Confirmation of ftshi3 T-DNA Insertion

As the *ftshi3-1 (kd)* phenotype differed from *ftshi3-2* and *FLAG_215F10*, an initial genotyping screening was performed on the GABI-KAT SET to confirm the T-DNA insertion (Supplementary Figure S1C). The entire genome of *ftshi3-1(kd)* was sequenced at the Beijing Genomics Institute (BGI, Denmark) to exclude additional T-DNA insertions (Supplementary Figure S1D). Two loci on Chr5 were partially penetrant when aligning the sequence of paired-end reads yielded from the WGS analysis, At5g09590, encoding MITOCHONDRIAL HSO702, and At5g09480, encoding a protein belonging to the hydroxyproline-rich glycoprotein family. Sanger sequencing of these loci in *ftshi3-1(kd)* revealed that they were intact (data not shown). Therefore, a single GABI-KAT T-DNA insertion in the *ftshi3-1(kd)* was confirmed.

**Figure 2:**
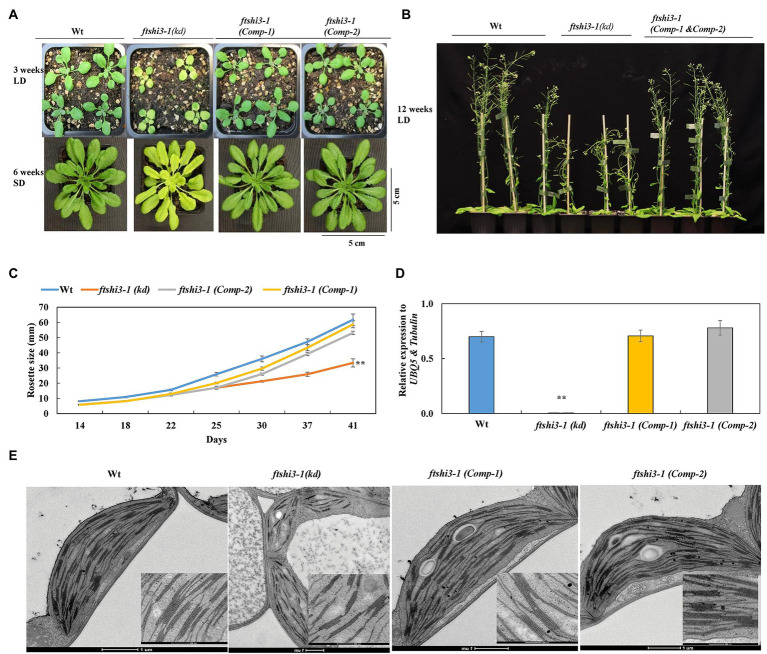
**(A)** Phenotypes of Wt, *ftshi3-1(kd)*, and *ftshi3-1* (*Comp-1* and *Comp-2*) at the age of 3 weeks grown in a long-day (LD; upper panel), 6 weeks in a SD (lower panel) or **(B)** 12 weeks in LD. **(C)** Rosette diameter measurements were performed from 2 to 6 weeks grown in SD. Asterisks indicate a significant difference (**p < 0.05, Student’s *t* test, *n* = 8). **(D)** *FTSHi3* gene expression analysis of Wt, *ftshi3-1(kd)*, and *ftshi3-1* (*Comp-1* and *Comp-2*) lines. Relative expression was normalized to *At-UBQ5* and *At-Tubulin*. Asterisks indicate a significant difference (**p < 0.05, Student’s *t* test, *n* = 3). Error bars represent the SE. **(E)** Transmission electron micrographs of chloroplasts of the first true leaves in Wt, *ftshi3-1(kd)*, and *ftshi3-1* (*Comp-1* and *Comp-2*) seedlings. Upper panel bars = 200 nm, lower panel bars = 1 μm.

Complementation was performed by expressing *FTSHi3* under the endogenous *AtFTSHi3* promoter in the *ftshi3-1(kd)* background. We designated the resulting transgenic *ftshi3-1 (Comp)* lines. Initially, 10 independent lines were screened in the T1 generation based on their phenotypes (Supplementary Figure S2A). Two representative transgenic lines in T3, *Comp-1*, and *Comp-2*, were selected for further investigations. Both lines displayed recovery to a Wt-like phenotype (Figures 2A,B). The diameters of eight independent rosettes per line measured from 2 to 6 weeks grown in the SD conditions were similar to Wt (Figure 2C). qPCR analysis on RNA extracted from 4-week-old plants grown in SD conditions confirmed restoration of *FTSHi3* transcript levels similar to Wt (Figure 2D).

The chloroplast ultrastructure in the first true leaves of *ftshi3-1(kd)* was disturbed with a compromised and thinner membrane; chloroplasts contained fewer and distorted thylakoid membranes and fewer starch granules (Figure 2E). Contrary to *ftshi3-1(kd)*, the chloroplast ultrastructure of the first true leaves of the *ftshi3-1(Comp)* lines revealed fully developed chloroplasts and thylakoid membranes similar to those of Wt (Figure 2E). PSII quantum yield (*Fv/Fm*) and NPQ performed on these plants grown in SD conditions were similar to Wt, while *ftshi3-1(kd)* displayed reduced *Fv/Fm* and significantly higher NPQ (Supplementary Figures S2B,C). The root growth phenotype and the number of lateral roots of *ftshi3-1(Comp-1 and Comp-2)* lines were similar to Wt (Supplementary Figures S2D,E,F).

MicroRNA lines were also generated expressing artificial miRNAs silencing *FTSHi3* expression. We designed miRNA silencing cassettes targeting the regions 533–553 (*miRNA-a*) and 1,952–1,972 (*miRNA-b*) of the *FTSHi3* coding sequence, respectively (Supplementary Figure S1B). Both cassettes were cloned downstream of a β-estradiol inducible promoter. T2 seedlings containing one of both silencing constructs displayed varying degrees of paleness, ranging from Wt-like plants to pale *ftshi3-1(kd)*-like plants (Supplementary Figure S3A). The paleness faded in adult plants, but they showed lowered overall growth throughout their life span (Supplementary Figures S3B; upper panel,C). *FTSHi3* transcript abundance was analyzed by extracting RNA from 14-day-old seedlings and 4-week-old plants (Supplementary Figures S3D,E). *FTSHi3* expression was downregulated in miRNA seedlings with the pale phenotype (*miRNA-a1-8* and *miRNA-b1-2, 5–6*), whereas those lacking paleness showed residual *FTSHi3* expression (Supplementary Figure S3C). *FTSHi3* expression was downregulated even in adult plants, showing the β-estradiol induction and silencing (Supplementary Figure S3E). These experiments collectively demonstrate that the lack of FtsHi3 in *ftshi3-1(kd)* is responsible for its distinct phenotype compared to the previously characterized *ftshi3* alleles.

### Loss of FtsHi3 Affects Stress Tolerance

**Figure 3:**
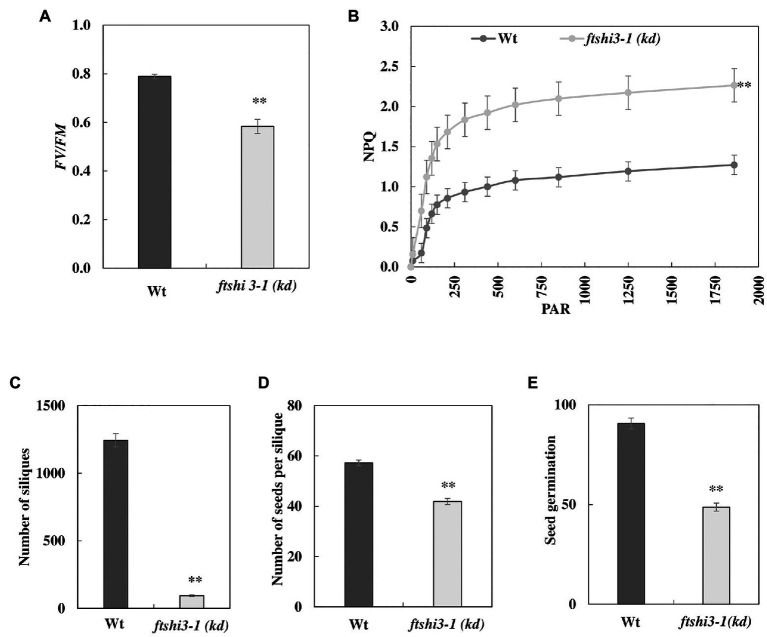
**(A)** Chlorophyll fluorescence indicating photosynthetic parameters (*Fv/Fm*) and **(B)** non-photochemical quenching (NPQ) of Wt and *ftshi3-1(kd)* measured on plants growing in the field. Asterisks indicate a significant difference (*p* < 0.05, Student’s *t* test, *n* = 8). The number of **(C)** siliques and **(D)** seeds per silique. Asterisks indicate a significant difference [^**^p < 0.001, ANOVA, least significant difference (LSD) *post hoc* test, *n* = 50]. **(E)** Seed germination capacity (determined after 2 days of incubation) of seeds harvested from field-grown plants. Asterisks indicate a significant difference (^**^p < 0.001, ANOVA, LSD *post hoc* test, *n* = 50). Error bars represent the SE.

As controlled laboratory growth conditions do not fully mimic the natural environment of plants, the seedlings were grown in a controlled growth chamber and field experiments were carried out under natural conditions in Umeå, Northern Sweden, in 2017. These studies were only performed on the transposon lines and not on the transgenic *ftshi3-1(Comp-1 and Comp-2)* lines due to restrictions in the permission. The absence of FtsHi3 yielded smaller plants than Wt in both the growth chamber and the field conditions (Figure 1C, lowest panel). Chlorophyll fluorescence parameters were determined on 45-day-old plants in the field. *Fv/Fm* was significantly lower in *ftshi3-1(kd)* compared to Wt (Figure 3A), whereas NPQ was significantly higher under the influence of increasing PAR (Figure 3B). Total chlorophyll contents and the chlorophyll *a/b* ratios of the seedlings were lower in the mutant (Supplementary Table S2).

We ended the field experiment when the first plants contained siliques that were almost dry and ready to shed their seeds. The total number of siliques was counted (Figure 3C), and further, the total number of seeds per silique was estimated by counting seeds from five siliques per plant (Figure 3D; Frenkel et al., 2008). Interestingly, all 50 *ftshi3-1(kd)* plants planted in the field survived throughout the field experiment despite their growth defects, compared to 43 Wt plants. However, *ftshi3-1(kd)* lines produced significantly lower numbers of siliques and seeds per silique than Wt (Figures 3C,D). Additionally, the germination of seeds harvested from field-grown *ftshi3-1(kd)* plants was strongly affected (Figure 3E), showing lower Darwinian fitness than Wt.

The more obvious phenotype of field-grown plants compared to plants grown under controlled conditions is caused by permanently varying growth conditions, including extreme light intensities (for short periods), temperature drops (day/night), flooding, and herbivore attacks (Mishra et al., 2012). To pinpoint which of these factors have the most potent effect on the growth of *ftshi3-1(kd)*, photochemical efficiency, and stress tolerance capacity, Wt and *ftshi3-1(kd)* plants were compared in laboratory conditions after subjecting them to the individual or combined stresses for 6, 9, and 16 weeks (if possible). At high temperature (30°C), continuous light or high light stress in SD, the rosette diameter of *ftshi3-1(kd)* increased compared to its growth in the respective control conditions (Supplementary Figures S4A–C,E). After 16 weeks of cold stress at SD conditions, *ftshi3-1(kd)* plants continued to display pale and small rosettes (Supplementary Figures S4D,E). Fluorescence quenching analyses showed significant differences in *Fv/Fm* and NPQ between *ftshi3-1(kd)* and Wt before exposure to stress (*t* = 0; Supplementary Table S3). *Fv/Fm* of *ftshi3-1(kd)* remained lower than Wt even after 3 days of stress exposure, showing slower stress acclimation (Supplementary Figure S4F; Supplementary Table S3). The *Fv/Fm* of *ftshi3-1(kd)* only recovered to Wt levels during growth at combined high temperature (30°C) and continuous light (Supplementary Table S3). The NPQ values of *ftshi3-1(kd)* increased and were significantly higher than those of Wt until 6 weeks of stress exposure (Supplementary Table S3), and dissipation of excess heat was better in the mutant than in Wt.

### ftshi3-1(kd) Plants Display Enhanced Tolerance to Drought Stress

**Figure 4:**
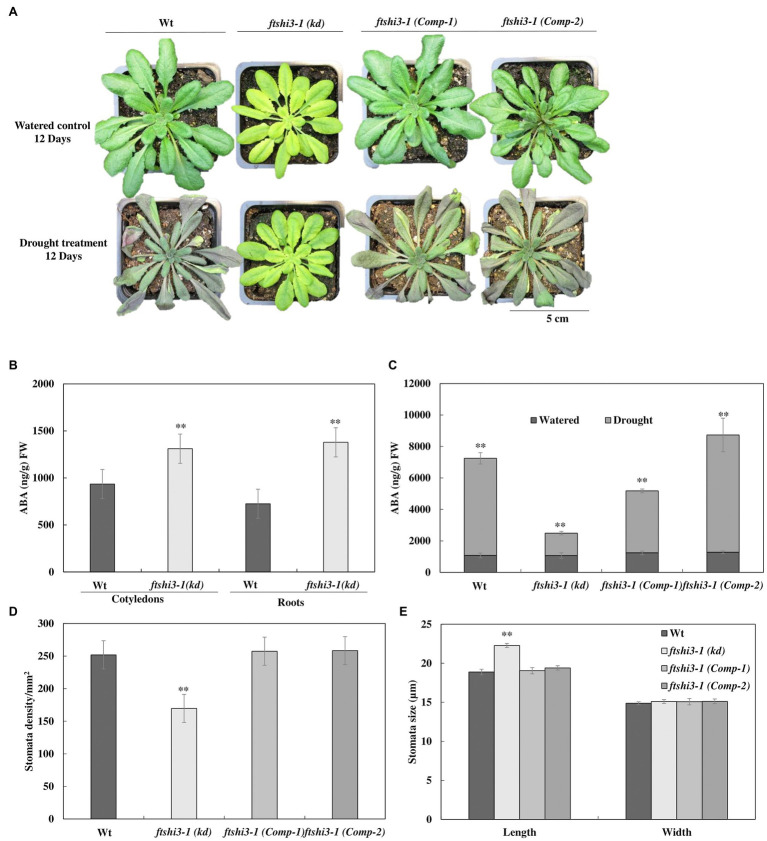
**(A)** Representative phenotypes of 6-week-old Wt, *ftshi3-1(kd)*, and *ftshi3-1 (Comp-1 and Comp-2)* plant after exposure to drought stress for 12 days. **(B)** Quantification of abscisic acid (ABA; ng/g) FW in 10-day-old seedlings of Wt and *ftshi3-1(kd)* grown in a growth chamber at LD and 22°C. Asterisks indicate a significant difference (**p < 0.05, Student’s *t* test, *n* = 3). Error bars represent the SE. **(C)** Quantification of ABA (ng/g) FW in leaves of watered and drought-stressed plants. Asterisks indicate the significant difference of ABA (ng/gm) content in watered and drought conditions (**p < 0.05, Student’s *t* test, *n* = 4). **(D)** Measurements of the stomatal density and **(E)** the stomatal size of Wt, *ftshi3-1(kd)*, and *ftshi3-1 (Comp-1 and Comp-2)*. Asterisks indicate a significant difference. Error bars represent the SE.

The performance of *ftshi3-1(kd)* was also tested under drought stress. Irrigation was stopped on 4-week-old, soil-grown plants until we observed lethal effects in over 50% of the genotype replicates under investigation. Wt and *ftshi3-1*(*Comp-1* and *Comp-2*) lines showed an early onset of drought sensitivity compared to *ftshi3-1(kd)* plants (Figure 4A, lower panel), while under controlled growth conditions with regular irrigation, all lines showed consistent growth (Figure 4A, upper panel). After 12 days of exposure to drought, almost all Wt and *ftshi3-1*(*Comp-1* and *Comp-2*) transgenic lines withered and became chlorotic, whereas 80% of the *ftshi3-1(kd)* plants continued to grow (Figure 4A), displaying no drought symptoms, although no difference in pot weight (g) was determined between the genotypes exposed to the same treatment (Supplementary Figure S5A). After 20 days of drought (Supplementary Figure S5B), the plants were re-irrigated with 350 ml of water every day for 14 days, and 15 random pots per tray were selected to test their survival rates (Supplementary Figure S5C). Over 80% of *ftshi3-1(kd)* plants recovered (Supplementary Figure S5C), whereas only 20% of Wt or *ftshi3-1*(*Comp-1* and *Comp-2*) lines survived. Wt, *ftshi3-1(kd)*, and *ftshi3-1* (*Comp-1* and *Comp-2*) lines were further grown in common trays over 2 weeks in LD conditions in the greenhouse (Supplementary Figure S5D); even shared reservoir *ftshi3-1(kd)* survived drought treatment for 15 days, while Wt and the complementation lines withered. The observed drought tolerance of *ftshi3-1(kd)* was independent of the growth time regime (Supplementary Figure S6).

Gas-exchange parameters during watered and drought conditions were determined under SD conditions (Table 1). No significant differences were observed in net photosynthesis (*AN*, μmol CO_2_ m^−2^ s^−1^) or CO_2_ concentration inside the leaf (Ci, μmol CO_2_ mol^−1^ air) across the genotypes. In contrast, the leaf stomatal conductance (*gs*, mol CO_2_ m^−2^ s^−1^) was significantly higher in *ftshi3-1(kd)* plants compared to Wt and *ftshi3-1*(*Comp-1* and *Comp-2*) in watered conditions. After 12 days of drought, Wt and *ftshi3-1*(*Comp-1* and *Comp-2*) had wilted; their *AN* and *gs*, therefore, were significantly lower compared to the values received for *ftshi3-1(kd)* plants (Table 1). The WUEi, i.e., the ratio *AN/gs*, showed a significant increase in *ftshi3-1(kd)* compared to Wt and *ftshi3-1*(*Comp-1* and *Comp-2*) during drought conditions (Table 1).

**Table 1:** Gas-exchange analyses performed on Wt, *ftshi3-1(kd)*, *ftshi3-1* (*Comp-1* and *Comp-2*), *miRNA-a2/a3/a5/a7*, and *Ftshi3-1*/*ftshi3-1* lines grown in watered and drought conditions.

	*An*		*gs*		*Ci*		*WUEi*	
	Watered	Drought	Watered	Drought	Watered	Drought	Watered	Drought
Wt	6.1 ± 0.4	0.8 ± 0.2	0.1 ± 0.0	0.0 ± 0.0	245.3 ± 4.2	263.6 ± 13.7	98.5 ± 4.2	90.1 ± 4.7
*ftshi3-1 (kd)*	6.0 ± 0.3	4.2 ± 0.2	0.1 ± 0.0	0.0 ± 0.0	312.4 ± 6.1	253.2 ± 2.4	46.1 ± 3.9	99.0 ± 1.5
*ftshi3-1 (Comp-1)*	6.2 ± 0.3	2.3 ± 0.4	0.1 ± 0.0	0.0 ± 0.0	221.8 ± 14.8	206.0 ± 18.2	85.1 ± 5.6	87.7 ± 8.5
*ftshi3-1 (Comp-2)*	6.0 ± 0.4	1.9 ± 0.1	0.1 ± 0.0	0.0 ± 0.0	232.5 ± 6.2	206.8 ± 19.2	95.9 ± 4.1	88.6 ± 6.8
*FTSHI3-1/ftshi3-1*	8.7 ± 0.4	5.2 ± 0.1	0.1 ± 0.0	0.1 ± 0.0	231.1 ± 3.9	235.8 ± 6.6	94.8 ± 2.7	90.3 ± 4.1
*miRNAa-2*	7.7 ± 0.1	6.4 ± 0.5	0.1 ± 0.0	0.1 ± 0.0	264.2 ± 1.4	225.6 ± 7.7	74.4 ± 0.9	91.1 ± 5.0
*miRNAa-3*	7.3 ± 0.1	5.1 ± 0.5	0.1 ± 0.0	0.1 ± 0.0	247.0 ± 1.4	249.0 ± 3.8	86.4 ± 0.9	85.1 ± 2.3
*miRNAa-5*	6.7 ± 0.2	7.9 ± 0.3	0.1 ± 0.0	0.1 ± 0.0	250.6 ± 2.2	267.5 ± 3.3	84.5 ± 1.6	73.0 ± 2.1
*miRNAa-7*	7.4 ± 0.3	7.2 ± 0.5	0.1 ± 0.0	0.1 ± 0.0	279.5 ± 3.1	261.4 ± 5.9	65.3 ± 2.1	76.8 ± 4.1

Values of net photosynthesis (*AN*, μmol CO_2_ m^−2^ s^−1^) and stomatal conductance (*gs*, mol CO_2_ m^−2^ s^−1^), CO_2_ concentration inside the leaf (*Ci*, μmol CO^2^ mol^−1^ air), and intrinsic water-use efficiency (WUEi). Values written in red indicate a *p*-value of less than 0.05. Student’s *t*-test was performed to determine the statistical significance. Error is SE.

Abscisic acid is an important hormone that increases plant tolerance to stressors (reviewed by Sah et al., 2016). To understand the responses to abiotic stress, the endogenous ABA levels in 10-day-old Wt and *ftshi3-1(kd)* seedlings grown in a growth chamber at LD and 22°C were determined. Cotyledons and roots of *ftshi3-1(kd)* seedlings showed a significantly higher ABA accumulation than Wt (Figure 4B). ABA levels were further investigated in watered and drought-stressed adult plants at the age of 6 weeks. While the levels of ABA (ng/g fresh weight) in the leaves of the adult watered plants were similar between lines (Figure 4C), ABA levels were significantly higher in Wt and *ftshi3-1*(*Comp-1* and *Comp-2*) than in *ftshi3-1(kd)* during drought stress (Figure 4C). Stomata density and sizes were determined in leaves of Wt, *ftshi3-1(kd)*, and *ftshi3-1(Comp-1 and Comp-2)* transgenic lines (Figures 4D,E). *ftshi3-1(kd)* plants contained an average of 169 stomata per mm^2^ as compared to Wt (252 per mm^2^) and the *ftshi3-1(Comp-1 and Comp-2)* lines (257 and 258 per mm^2^, respectively; Figure 4D). The average stomatal width to length dimension was 18.9 by 14.9 μm for Wt, in contrast to 22.3 by 15.1 μm for *ftshi3-1(kd)* and 19.05 by 15.08 μm or 19.1 by 15.13 μm for the *ftshi3-1*(*Comp-1* and *Comp-2*) lines, respectively (Figure 4E). The differences observed between the length of the stomata and the stomata density per mm^2^ in *ftshi3-1(kd)* compared to Wt and the *ftshi3-1(Comp-1 and Comp-2)* lines were significant (Figure 4E).

**Figure 5:**
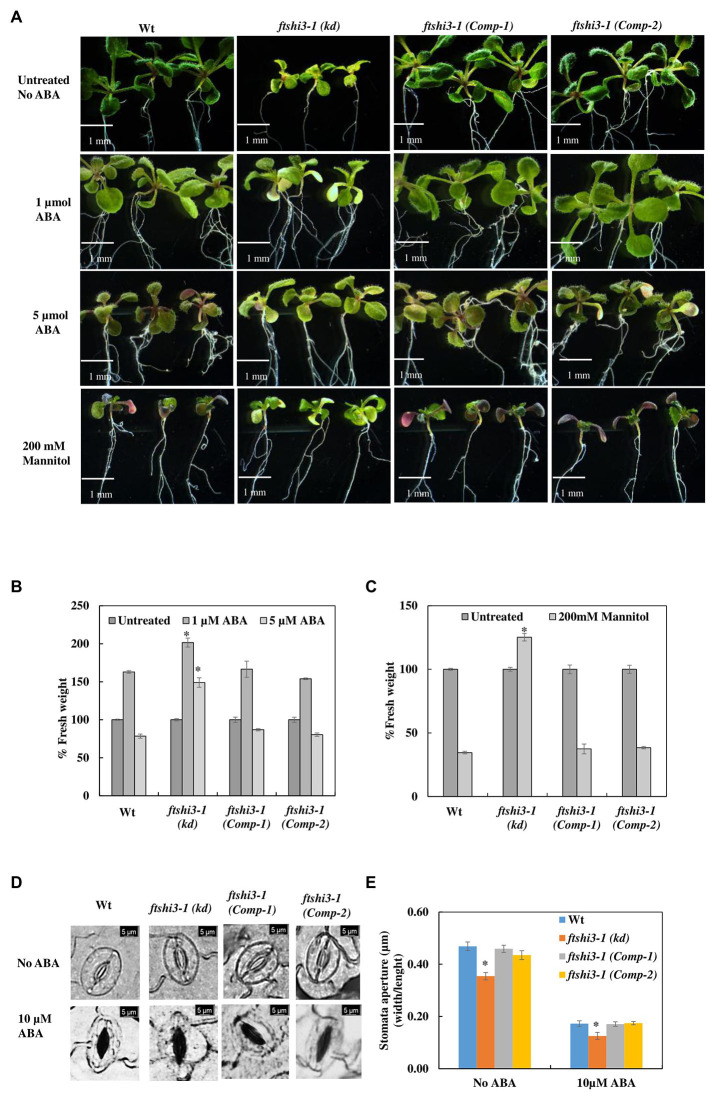
**(A)** Seedlings of Wt, *ftshi3-1(kd)*, and *ftshi3-1 (Comp-1 and Comp-2)* grown in the absence of exogenous ABA and mannitol or exposed to 1or 5 μM ABA or 200 mM mannitol. **(B,C)** Quantification of fresh weight after treatment with 1 or 5 μM ABA or 200 mM mannitol. Asterisks indicate the significant difference to Wt (*p < 0.05, Student’s *t* test, three sets containing 15 biological replicates each per treatment per genotype). Scale bar: 1 mm. Error bars represent the SE. (**D)** Microscopic images of stomatal closure in the presence or absence of 10 μM ABA in Wt, *ftshi3-1(kd)*, and *ftshi3-1 (Comp-1 and Comp-2)* plants. Scale bar: 5 μm. **(E)** Graphical presentation of the stomatal closure in the presence or absence of 10 μM ABA in Wt, *ftshi3-1(kd)*, and *ftshi3-1 (Comp-1 and Comp-2)* plants. Asterisks indicate significant differences (Student’s *t* test, *p < 0.05, five biological replicates; 45 stomata per leaf). Error bars represent the SE.

Abscisic acid has several implications on overall plant growth and development, including regulating plant water balance and osmotic stress tolerance (Cutler et al., 2010); therefore, treatment with exogenous ABA is a way of regulating plant tolerance in agriculture (reviewed by Sah et al., 2016). We investigated the effect of mannitol-induced osmotic stress and exogenous ABA treatment to understand the responses to dehydration and abiotic stress tolerance in Wt, *ftshi3-1(kd)*, and *ftshi3-1 (Comp-1 and Comp-2)* seedlings. Seven-day-old seedlings of Wt, *ftshi3-1(kd)*, and *ftshi3-1(Comp-1 and Comp-2)* were grown either in the absence of exogenous ABA and mannitol or exposed to 1 or 5 μM ABA, or 200 mM mannitol for another 7 days. Seedlings of *ftshi3-1(kd)* sustained significantly in growth rate compared to Wt and the Comp lines when treated with either ABA or mannitol (Figures 5A–C). We further examined the *ftshi3-1(kd)* guard cell apertures at the abaxial side of the leaves compared to Wt and the *ftshi3-1(Comp-1 and Comp-2)* lines to understand if the stomatal aperture closure is sensitive to ABA (Figures 5D,E). Response to ABA was determined by calculating the ratio of width/length of the stomata in the presence or absence of exogenous 10 μM ABA (Figures 5D,E). Stomatal apertures in the absence of ABA treatment were smallest in *ftshi3-1(kd)*, averaging 0.35 μm, whereas they averaged 0.47 μm in Wt, and 0.46 and 0.43 μm in the *ftshi3-1(Comp-1 and Comp-2)* lines, respectively (Figure 5E). When treated with 10 μM exogenous ABA, stomatal closure was induced, with the stomatal apertures of all genotypes decreasing by over 50% (Figure 5E). These results suggest that drought tolerance in *ftshi3-1(kd)* plants is related to their lower stomatal density instead of ABA sensitivity.

### Transcript Abundance of ABA-Responsive Genes in ftshi3-1(kd) Plants

**Figure 6:**
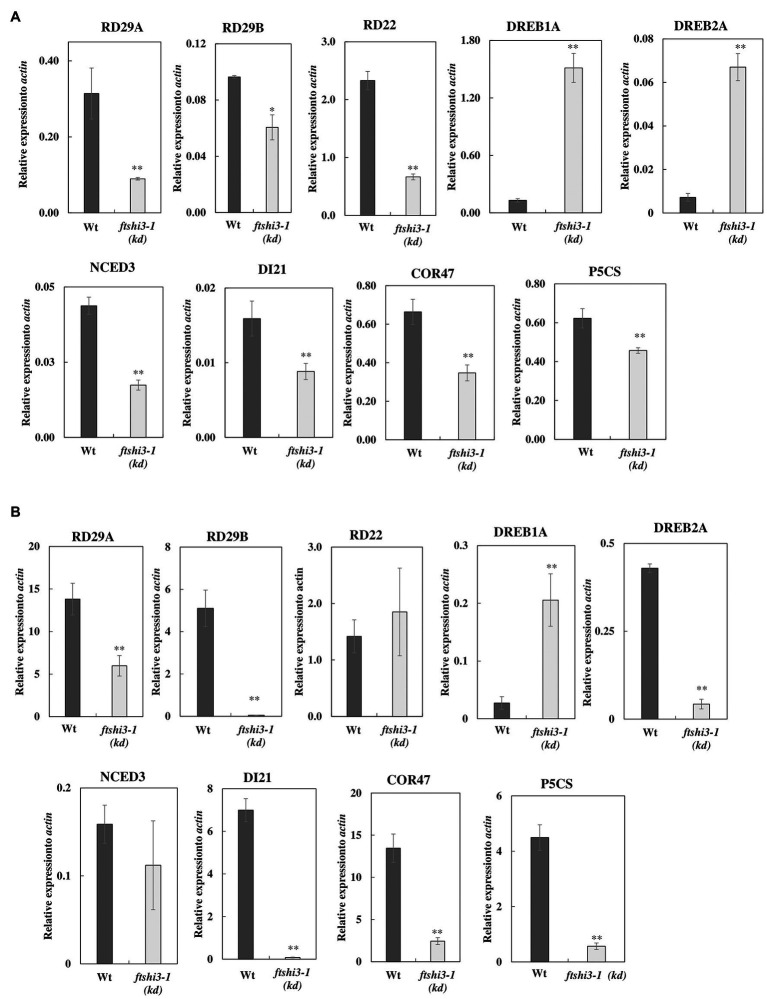
**(A)** Relative expression of drought-responsive genes in *ftshi3-1(kd)* in watered conditions. The data were normalized to the expression of genes coding for *Arabidopsis*, *actin*. Asterisks indicate significant differences (Student’s *t* test, *p < 0.05; **p < 0.001, *n* = 3). **(B)** Relative expression of drought-responsive genes under drought conditions for 14 days, normalized to the expression of *actin*. Asterisks indicate significant differences (Student’s *t* test, **p < 0.001, *n* = 3). Error bars represent the SE.

Drought stress activates ABA-biosynthesis, leading to the expression of ABA-responsive genes, which can be signaled either *via* an ABA-dependent or an ABA-independent pathway or by cross-talk between both pathways (Yoshida et al., 2014). Among several studied ABA-responsive genes (reviewed by Shinozaki and Yamaguchi-Shinozaki, 2006; Ahuja et al., 2010), we investigated RESPONSIVE TO DESICCATION 29A, 29B, and 22 (*RD29A*, *RD29B*, *RD22*), Dehydration-responsive element-binding protein 1A and 2A (*DREB1A*, *DREB2A*; Liu et al., 1998; Nakashima et al., 2009), 9-cis-epoxycarotenoid dioxygenase (*NCED3*, a key enzyme in the biosynthesis of ABA; Iuchi et al., 2001), and ABA-regulated gene *COR47* (COLD-REGULATED 47) belonging to the dehydrin protein family. We also investigated genes whose expression indicates progressive drought stress, *DI21* (DROUGHT-INDUCED21; Gosti et al., 1995) and *P5CS* (delta1-pyrroline-5-carboxylate synthase), which is involved in the biosynthesis of proline but is expressed in the whole plant under water-limiting conditions (Yoshiba et al., 1999). The transcript accumulation of ABA-responsive genes was determined in both watered (Figure 6A; Supplementary Figure S7A) and drought (Figure 6B; Supplementary Figure S7B) conditions in Wt, *ftshi3-1(kd)*, and in the *ftshi3-1(Comp-1 and Comp-2)* lines. The expression of *RD29A*, *RD29B*, *RD22*, *DREB1A*, *DREB2A*, *NCED3*, *DI21*, *COR47*, and *P5CS* in *ftshi3-1(kd)* and Wt in watered condition (Figure 6A) and drought conditions (Figure 6B) are shown in relation to the housekeeping gene *actin*. Under watered conditions, the expression of two ABA-independent genes, *DREB1A* and *DREB2A*, were significantly higher in *ftshi3-1(kd*; Figure 6A). In comparison, *DREB1A*, but not *DREB2A*, was significantly upregulated during drought in *ftshi3-1(kd)* plants relative to Wt (Figure 6B). The expression of other drought-responsive genes *RD29A*, *RD29B*, *RD22*, *COR47*, *NCED3*, and *P5CS* were significantly lower in *ftshi3-1(kd)* plants compared to Wt in watered and drought conditions. Although the underlying mechanism for these expression patterns is unknown, *RD29A*, *RD29B*, *RD22*, *NCED3*, *DI21*, *COR47*, and *P5CS* are also markers of progressive drought; except for *RD22*, all these genes were significantly upregulated in the drought-sensitive Wt compared to *ftshi3-1(kd)* plants after 12 days of drought (Figure 6B). Expression of *RD22*, *RD29B*, *NCED3*, *DI21*, and *P5CS* in the *ftshi3-1(Comp-1 and Comp-2)* lines was comparable to Wt when grown in either condition (Supplementary Figures S7A,B). These data corroborate our observation that ABA accumulation in *ftshi3-1(kd)* plants is elevated in watered seedlings and reduced during drought (Figures 4B,C).

### MicroRNA Induced Silencing of FtsHi3 Also Generates a Drought-Tolerant Phenotype

We performed drought stress experiments on *miRNA-a2/a3/a5/a7* chosen based on their low expression of *FTSHi3* (Supplementary Figure S3B). Similar to *ftshi3-1(kd)* plants, *miRNA-a2/a3/a5/a7* plants were drought tolerant (Supplementary Figure S3B, lower panel). Additionally, similar to *ftshi3-1(kd)*, the expression of DREB1A and DREB2A was enhanced in *miRNA-a2/a3/a5/a7* plants compared to Wt in watered conditions (Supplementary Figures S3F,G).

We also investigated heterozygous *ftshi3-1* T-DNA lines (*FTSHi3-1*/*ftshi3-1*; Supplementary Figure S8A). *FTSHi3-1*/*ftshi3-1* lines were genotyped from the batch of seeds obtained from NASC. Similar to *ftshi3-1(kd)* and *miRNA-a2/a3/a5/a7*, the heterozygous lines displayed reduced rosette size, lowered *FTSHi3* expression similar to the miRNA lines, and non-pale older plants (Supplementary Figures S8A,C–D). When exposed to drought stress, *FTSHi3-1*/*ftshi3-1* lines behaved like *ftshi3-1(kd)* and *miRNA-a2/a3/a5/a7* plants (Supplementary Figures S3B, S8A; Table 1), suggesting that the pleiotropic association of *FTSHi3* expression to drought tolerance is not necessarily linked to the pale green phenotype.

### ftshi3 (kd) Plants Recruit a Differential Drought-Responsive Bacterial Community Compared to Wt

**Figure 7:**
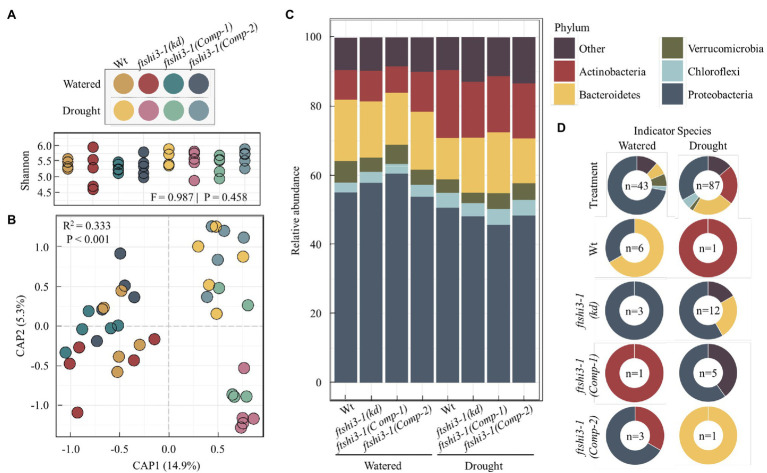
The root-associated bacteria of *ftshi3-1(kd)* shift under drought. **(A)** Alpha diversity (Shannon) of root-associated bacteria. Colors depict different watering treatments and host genotypes. **(B)** Constrained ordination of root-associated bacterial community composition constrained the interaction between treatment and genotype and colored as in **(A)**. **(C)** The relative abundance of root bacteria at the phylum level. Colors represent the top five phyla. Additional phyla combined as “Other.” **(D)** Significant bacterial indicators of watering or drought treatment across all genotypes (top two plots) or between all genotypes and treatments (bottom eight plots) identified by Dufrene-Legendre analysis (*p* < 0.05, indcls > 0.5). The number of significant indicators identified for each group is listed in the center of each chart. Indicators were merged by phyla, with colors representing the top five phyla as in **(C)**.

*ftshi3-1(kd)* plants display enhanced drought tolerance, reduced root length, and reduced lateral root formation. To test if these phenotypes result in the colonization of distinct root-associated bacteria, watered and drought treatments were applied to Wt, *ftshi3-1(kd)*, and *ftshi3-1(Comp-1 and Comp-2)* grown in “common garden” trays with a shared soil reservoir. Roots were harvested, and we performed 16S rRNA community profiling. Alpha diversity between genotypes did not vary significantly by treatment (Shannon, *F* = 0.987, *p* = 0.458; Figure 7A). However, CAP of Bray-Curtis distances constrained for the interaction between treatment by genotype showed a significant shift in beta diversity (*R*^2^ = 0.333, *p* < 0.001), with CAP1 separating samples by watering treatment (Figure 7B). Pairwise PERMANOVA calculated the separation between samples. Notably, while the aboveground phenotypes of *ftshi3-1(kd)* are consistent with increased drought tolerance, both *ftshi3-1(kd)* and Wt recruited significantly different bacterial profiles between watering and drought treatments (Wt, *R*^2^ = 0.233, *Q* = 0.045; *ftshi3-1(kd)*, *R*^2^ = 0.273, *Q* = 0.023), suggesting that *ftshi3-1(kd)* plants were responding to water stress (Figure 7B; Supplementary Table S4). Further supporting this observation, all genotypes displayed increases in the relative abundance of Actinobacteria under drought (Figure 7C), consistent with drought response in roots (Naylor et al., 2017; Fitzpatrick et al., 2018). While all genotypes demonstrated an increase in the relative abundance of Actinobacteria, drought contributed to distinct bacterial communities between Wt and *ftshi3-1(kd; R*^2^ = 0.201, *Q* = 0.028; Figure 7B; Supplementary Table S4). We performed indicator species analyses to identify genera associated with different watering treatments and/or host genotypes. We identified 43 significant indicators of watered conditions and 87 associated with drought (Dufrene-Legendre, *p* < 0.05, indcls > 0.5; Figure 7D; Supplementary Table S5). Consistent with the increase in the overall relative abundance of Actinobacteria during drought, all significant indicators belonging to the phyla Actinobacteria were associated with drought (Figure 7D). Compared to watering vs. drought treatments, fewer bacterial indicators were identified for different host genotypes. For example, only a single indicator belonging to the phyla Actinobacteria (*Arthrobacter* sp.) was found to be explicitly associated with Wt during drought, and none were explicitly associated with *ftshi3-1(kd)*, whereas 19 Actinobacteria indicators were broadly associated with drought treatment rather than a specific host genotype (Figure 7D). However, the drought *ftshi3-1(kd)* had the largest number of significant indicators of any genotype (*n* = 12; Figure 7D; Supplementary Table S5), suggesting it harbored the most distinct bacterial community. These data collectively suggest that *ftshi3-1(kd)* displays drought-tolerant phenotypes in aboveground tissue, while the root-associated bacterial community responds to drought.

## Discussion

The chloroplast serves as the compartment for photosynthesis and houses, many vital metabolic pathways crucial for various aspects of plant growth and development (Sakamoto et al., 2008). Plants carrying a mutation in a nuclear-encoded, plastid-localized protein, therefore, often display pale-seedling phenotypes (Myouga et al., 2010). Seedlings with reduced amounts or depleted in one of the FtsHi pseudo-protease family members are commonly pale (Kadirjan-Kalbach et al., 2012; Lu et al., 2014; Kikuchi et al., 2018; Schreier et al., 2018; Wang et al., 2018c; Mishra et al., 2019; Schäfer et al., 2019). Contrary to earlier described *ftshi3* mutants (Kikuchi et al., 2018; Mishra et al., 2019), *ftshi3-1(kd; GK-555D09-021662)* showed not only a pale seedling phenotype but also reduced root growth and significant growth reduction through its life span (Figures 1A–G). Chloroplasts of *ftshi3-1(kd)* plants had aberrant chloroplast ultrastructure. The question, therefore, arises of why the phenotype of *ftshi3-1(kd)* differs from the ones of *ftshi3-2* (Mishra et al., 2019) and *FLAG_215F10* (Kikuchi et al., 2018). In *ftshi3-1(kd)*, *FTSHi3* transcription was almost absent (Figure 1B), while an expression of 10% remained in *ftshi3-2* (Mishra et al., 2019). Therefore, one could speculate that relatively high amounts of FtsHi3 per cell are needed during early seedling development, leading to a pale seedling phenotype even in mutants with moderate expression of the enzyme. However, in adult plants, the amount of FtsHi3 needed for normal development is lower, supporting this hypothesis, and both *ftshi3-2* (Mishra et al., 2019) and *FLAG_215F10* (Kikuchi et al., 2018) develop into Wt-looking plants. Similarly, our miRNA and heterozygous lines displayed Wt-looking adult plants, while the growth reduction and pale phenotype of *ftshi3-1(kd)* persist throughout its lifespan.

Another possible explanation of the differences in phenotypes could be the site of transposon insertion (Supplementary Figure S1A). A C2H2(Zn) transcription factor binding site (TFBS) is located in the upstream region of the *FTSHi3* gene and at the exact location of the *ftshi3-1(kd)* T-DNA insertion site (highlighted in Supplementary Figure S1A). PlantPAN 3.0 (Chow et al., 2018) and AthaMap (Hehl et al., 2015) both predicted TFBS for the ZAT members ZAT10, ZAT18 (indicated by red and black triangles), and ZAT2 (indicated by orange circles triangles) on the 5′UTR. C2H2-EAR zinc finger protein Zat10 in *Arabidopsis* enhances the tolerance of plants to osmotic stress (Mittler et al., 2006). ZAT10 is required for responses to abiotic stress (reviewed by Ciftci-Yilmaz and Mittler, 2008; Han et al., 2020) and can act as both activator and repressor of stress-response genes, especially during drought or osmotic stress (Mittler et al., 2006). We are aware that this TFBS is located within the coding region, but knowledge on TFs is progressing and might provide a probable explanation for the variation of the phenotype between *ftshi3-1* (*GK-555D09-021662*) and *ftshi3-2* (GK-723C06_025364) and *ftshi3* (FLAG_215F10).

Growth under (semi-)natural conditions is challenging for plants and might affect their Darwinian fitness. While *ftshi3-1(kd)* could survive the stress arising from challenging field conditions (all 50 plants growing in the field survived), seed dormancy and germination indicated lower Darwinian fitness (Figures 3D,E). Plant hormones like ABA and gibberellins (Finch-Savage and Leubner-Metzger, 2006; Rodríguez-Gacio Mdel et al., 2009) influence seed dormancy and germination; for efficient germination, ABA levels should remain low in germinating seeds (Wang et al., 2018a). *ftshi3-1(kd)* seedlings (cotyledons and roots) showed significantly higher accumulation of ABA compared to Wt (Figure 4B); the delay in their germination, therefore, might be caused by overaccumulation of ABA. Since elevated ABA enhances the adaptation of plants to various abiotic stresses (Tuteja, 2007), *ftshi3-1(kd)* plants might be primed to handle abiotic stress better than the Wt (Gunapati et al., 2016; Liu et al., 2019).

Most intriguing was the drought tolerance of *ftshi3-1(kd)*. Exposed to drought stress, the performance of *ftshi3-1(kd)* was far better than Wt, and the mutant showed signs of drought only after 20 days (Figures 4A,C; Supplementary Figure S5A). *ftshi3-1(kd)* plants are smaller than Wt; however, we do not believe that the drought tolerance of this mutant is caused only by less water usage. *ftshi3-1(kd)* displayed drought tolerance also in shared and common trays with Wt (e.g., Supplementary Figures S5B,C) or in “common garden” experiments with soil and water as a shared reservoir between Wt and *ftshi3-1(kd*; Supplementary Figure S5D). The *ftshi3-1(kd)* mutant senses drought, as shown by its specific root-associated microbiome. Drought stress has been shown to enrich *Actinobacteria* within the root microbiome of a broad range of angiosperms (Naylor et al., 2017; Fitzpatrick et al., 2018). Therefore, drought-tolerant *ftshi3-1(kd)* were predicted to display reduced enrichment of *Actinobacteria* during drought stress compared to Wt. However, the community composition of all genotypes significantly differed between watering treatments, and similar increases in the relative abundance of *Actinobacteria* were observed across all genotypes (Figure 7). Furthermore, 95% of drought-associated *Actinobacteria* identified by indicator species analysis were genotype agnostic (Supplementary Table S5). These data suggest that, while above ground phenotypes of *ftshi3-1(kd)* are consistent with increased drought tolerance, the root-associated bacterial community of *ftshi3-1(kd)* during drought treatment responded to water stress.

While our data support a typical drought response of *ftshi3-1(kd)*, the bacterial community composition differed from Wt, particularly during drought. Notably, ABA, which plays an essential role during drought stress tolerance in plants and also antagonises the plant immune system *via* a salicylic acid-dependent mechanism (Yasuda et al., 2008). Changes in salicylic acid biosynthesis and signaling are known to modulate root-associated colonization of bacteria (Lebeis et al., 2015). We propose that the distinct bacterial community observed in *ftshi3-1(kd)* during drought is driven by significant differences in ABA accumulation and regulation compared with Wt. If and how these distinct root-associated bacteria contribute to the increased drought tolerance observed in *ftshi3-1(kd)* will be an interesting avenue for future research.

Abscisic acid concentrations were significantly lower in *ftshi3-1(kd)* exposed to drought stress than Wt or the complementation lines, indicating a delay in perceiving drought stress above ground. Increased endogenous ABA concentrations are common for drought-stressed plants with closed stomata to prevent intracellular water loss (Radin and Ackerson, 1981; Cutler et al., 2010). Osmotic stress imposed by drought was shown to be transmitted either *via* an ABA-dependent or an ABA-independent pathway (Yoshida et al., 2014). DREB1A/DREB2A plays an essential role in the ABA-independent acclimation to drought stress (Nakashima et al., 2009). DREB1A and DREB2A are transcription factors that recognize the *cis*-acting element DRE/CRT, which is involved in gene expression during dehydration (Liu et al., 1998; Nakashima et al., 2009). Among the drought-responsive genes investigated, *DREB1A* was significantly higher expressed in *ftshi3-1(kd)* in both watered and drought conditions, while *DREB2A* was transcribed to a greater extent only in watered conditions (Figures 6A,B). Drought-tolerance of *ftshi3-1(kd)*, therefore, seems to be regulated independently of ABA. Overexpression of *DREB1A/DREB2A* causes a dwarfed phenotype in transgenic plants (Liu et al., 1998), which is confirmed by *ftshi3-1(kd)* expressing *DREB1A* and *DREB2A* at least four times as high as Wt. The lower transcription of genes associated with progressive drought in *ftshi3-1(kd)* and the lower accumulation of ABA compared to the drought-sensitive Wt and complementation lines point toward a significant delay of *ftshi3-1(kd)* plants to sense dehydration above ground. While the lower stomatal density and size is accountable, the decreased stomatal conductance in *ftshi3-1(kd)* and/or other mechanisms is still to be explored.

Mannitol-induced osmotic stress and treatment with exogenous ABA in seedlings enabled us to understand the responses to abiotic stress at the seedling stage of leaf development (Figures 5B,C). Despite the high natural concentration of ABA in *ftshi3-1(kd)* seedlings, they responded positively to exogenous ABA treatments and survived better than Wt (Figure 5B); *ftshi3-1(kd)*, therefore, is ABA sensitive. Effects observed on plants after exogenous ABA treatment differ from those induced by endogenous ABA (Okamoto et al., 2010). While *ftshi3-1(kd)* was able to grow (increase in % of fresh weight) at a concentration of 5 μM exogenous ABA, growth of Wt and the complementation lines was inhibited (Figures 5A–C). This dose- and developmental stage-dependent growth response of *ftshi3-1(kd)* points toward a semi-sensitivity to ABA (Li et al., 2017; Rosales et al., 2019).

We are the first to show an *Arabidopsis* mutant with reduced expression of the FtsHi3 pseudo-protease to be drought-tolerant. Detailed analysis of this mutant revealed the accumulation of a drought-specific root-associated microbiome. Enhanced *FTSH* (but not *FTSHi*) expression during drought was observed in *Zea mays*. *ZmFTSH6* (Zm00001d037232) was upregulated in the drought-tolerant maize mutant (C7–2t; Zhang et al., 2020); however, transgenic tobacco overexpressing ZmFtsH2A and ZmFtsH2B was found not to be drought-tolerant (Yue et al., 2010). Reduced gene expression of *FtsH2* and an *FtsH-like* was observed in *Solanum lycopersicum* L. in response to drought stress (Tamburino et al., 2017). A reduced abundance of FtsH proteins was recognized in a susceptible Kentucky bluegrass cultivar exposed to drought stress (Xu and Huang, 2010). Comparing the proteomes of Tibetan wild and cultivated genotypes (drought-tolerant XZ5 and drought-sensitive XZ54 and cv. ZAU3) exposed to drought stress identified the amount of the chloroplast thylakoid-located FtsH1 to remain unchanged in XZ5, while it was diminished in XZ54 and ZAU3 (Wang et al., 2015). Although the molecular mechanisms remain unclear, our findings suggest that *ftshi3-1(kd)* displayed a drought-tolerant phenotype both in young and adult plants in aboveground tissue, while the root-associated bacterial community responded to drought.

## Data Availability Statement

The datasets presented in this study can be found in online repositories. The names of the repository/repositories and accession number(s) can be found in the article/**Supplementary Material**.

## Author Contributions

LM, SM, and DC performed the experiments, involved in the formal analysis, and characterized the mutant plants. DC-D and CF supervised the project. CF conceptualized the project and was responsible for funding acquisition. All authors were involved in writing the manuscript. All authors contributed to the article and approved the submitted version.

### Conflict of Interest

The authors declare that the research was conducted in the absence of any commercial or financial relationships that could be construed as a potential conflict of interest.

## Footnotes

^1^http://www.bioinformatics.babraham.ac.uk/projects/fastqc/
