# Supplementary material for: The Plastid-Localized AtFtsHi3 Pseudo-Protease of Arabidopsis thaliana Has an Impact on Plant Growth and Drought Tolerance
Source: Front Plant Sci. 2021 Jun 23;12:694727. doi: 10.3389/fpls.2021.694727 (PMC8261292; doi:10.3389/fpls.2021.694727)
Supplement: Supplementary file 2 [file Data_Sheet_1.DOCX]

Supplementary Material

**The plastid-localised AtFtsHi3 pseudo-protease of *Arabidopsis thaliana* has an impact on plant growth and drought tolerance**

**Laxmi S. Mishra^a^, Sanatkumar Mishra^b1^, Daniel Caddell^c1^, Devin Coleman-Derr^c,d^ and Christiane Funk^a^**

**Corresponding author: Christiane Funk** [***christiane.funk@umu.se***](mailto:christiane.funk@umu.se) **Tel: +46(0)907867633**


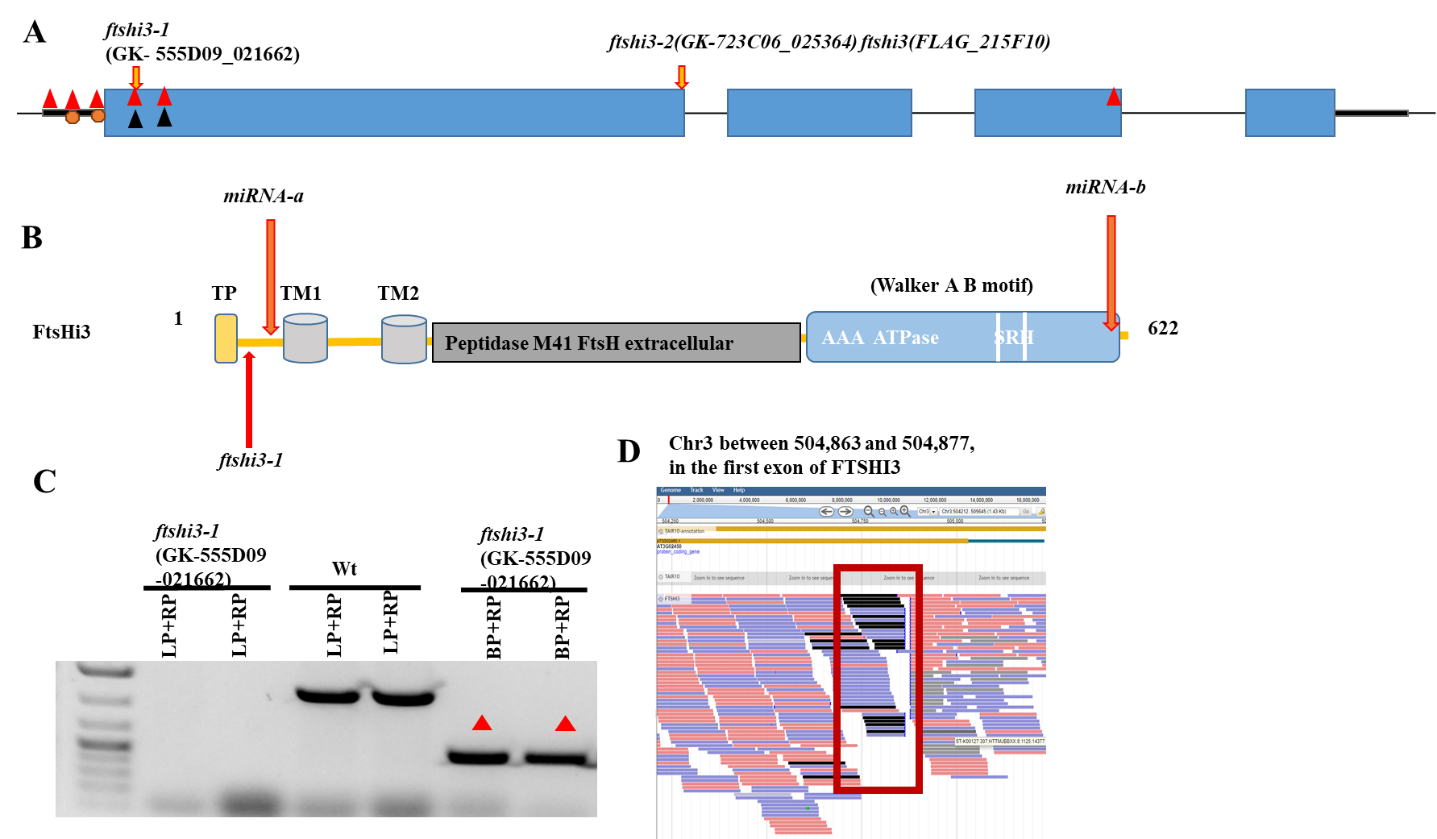


**Figure S1**: **(A)** Schematic diagram of the *FTSHi3* gene, indicating T-DNA insertion locations generating *ftshi3-1* and *ftshi3-2* (orange arrows). Highlighted in black are the UTRs; red triangles indicate the position of ZAT 10 motifs predicted by PlantPAN 3.0. Black triangles and orange circles indicate ZAT18 and ZAT2 transcription factor motifs predicted by AthaMap. **(B)** Schematic diagram of FtsHi3 indicating the mutations of miRNA-a *miRNA-b* and the location of the T-DNA insertion (*ftshi3-1*). *miRNA-a* and *miRNA-b* indicate the regions of modification of micro-RNA lines. **(C)** Agarose gel after genotyping of *ftshi3-1* *(GK-555D09-021662)* by PCR, left (LP) and right (RP) genomic primers as well as T-DNA border primer (BP) are given in Supplementary Table 1. **(D)** JBrowse screenshot showing the TAIR 10 version of the *Arabidopsis* genome as a reference, with the paired-end reads aligned to it. The red box highlights the T-DNA insertion site on Chr3 between bp 504,863 and 504,877, in the first exon of *FTSHi3* (At3g02450).


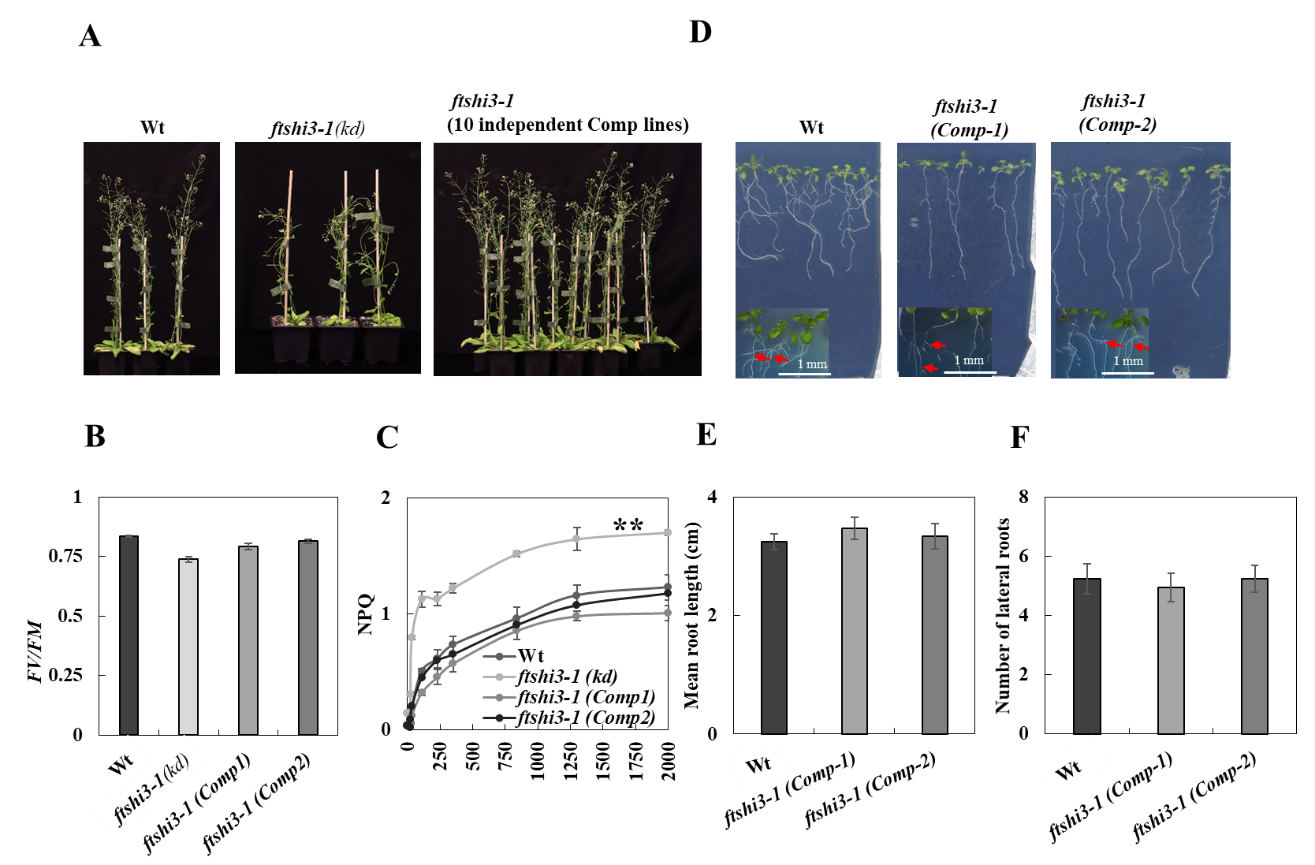


**Figure S2. (A)** Phenotypes of Wt, *ftshi3-1(kd)* and *ftshi3-1 Comp* (10 independent complementation lines) at the age of 12 weeks grown at LD. **(B)** Chlorophyll fluorescence measurements are indicating the photosynthetic parameters (*Fv/Fm*) and **(C)** non-photochemical quenching in Wt, *ftshi3-1(kd)*  and *ftshi3-1 (Comp-1* and *Comp-2)*. Asterisks indicate a significant difference (P< 0.05, Student’s t-test, eight biological replicates represented). **(D)** Seedling root phenotypes of Wt and *ftshi3-1 (Comp-1* and *Comp-2)* at the age of 8 days. Arrows indicate the lateral roots. **(E)** Graphical representation of the root length (cm) and **(F)** number of lateral roots (n > 20).


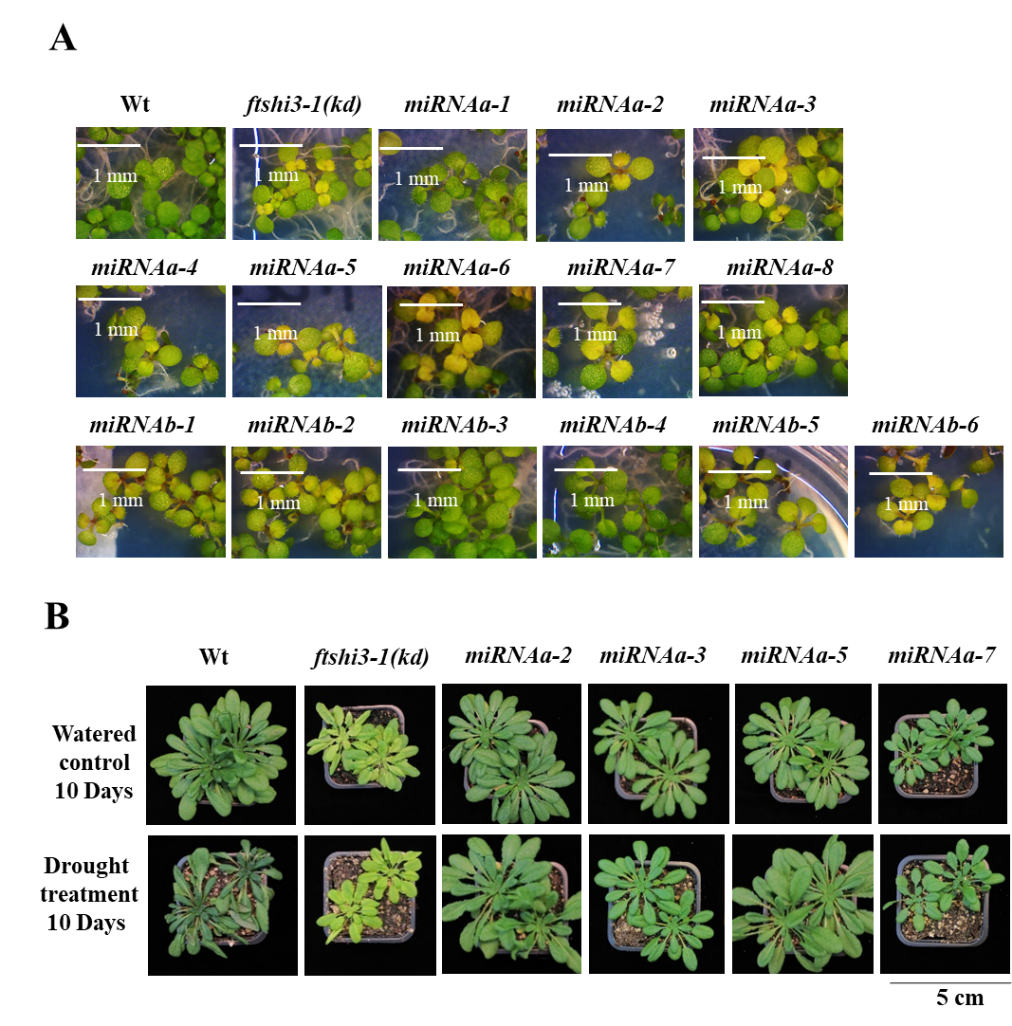


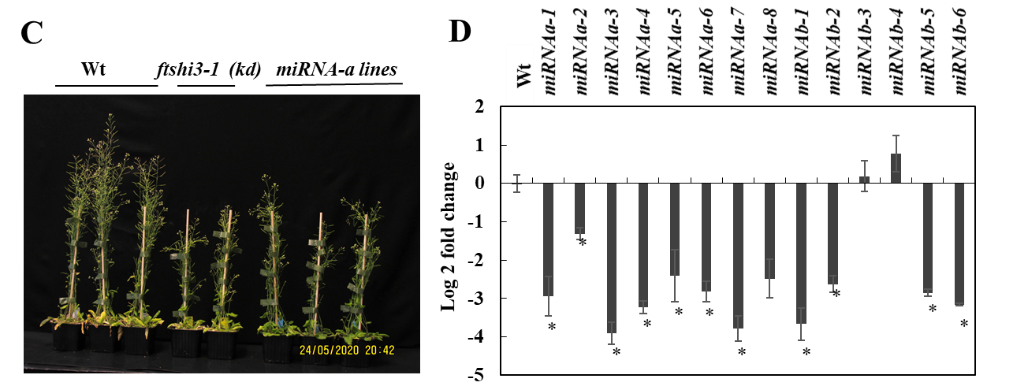


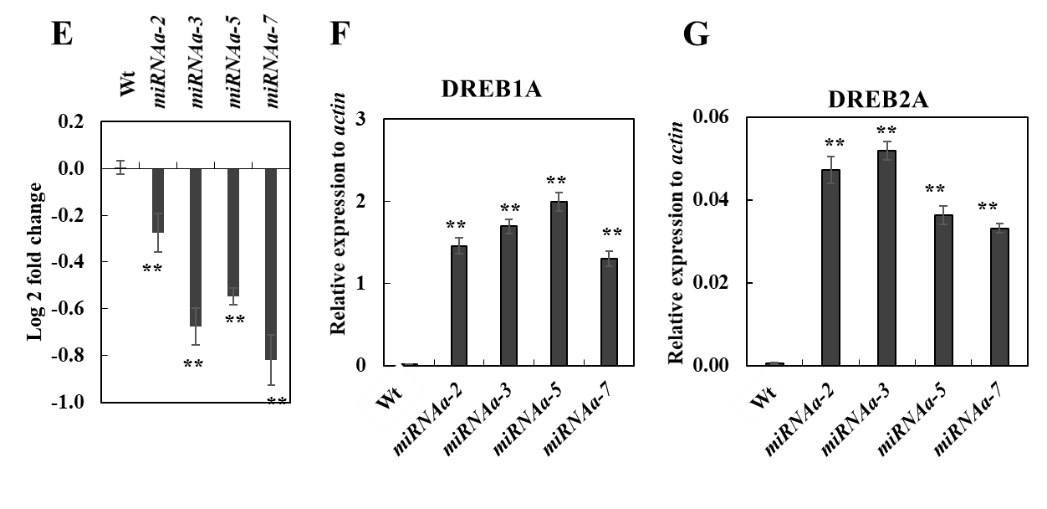


**Figure S3**: **(A)** Seedling phenotype of Wt*, ftshi3-1(kd)*, and the miRNA lines *miRNA-a* and *miRNA-b*. **(B)** Representative phenotypes (from eight replicates) of four-week-old plants of Wt*, ftshi3-1(kd)* and *miRNA-a2/a3/a5/a7* under watered conditions (upper panel) or exposed to drought stress (lower panel). **(C)** Phenotypes of Wt, *ftshi3-1(kd)* and *miRNA-a* lines (3 representative lines) at the age of 12 weeks grown in LD. **(D)** Transcript abundance of *FTSHi3* in seedlings of Wt and the *miRNA-a* and *miRNA-b* lines. Asterisks indicate significant differences (Student’s t-test, P< 0.05, n=3). Error bars represent the SE. **(E)** Transcript abundance of *FTSHi3* in four-week-old Wt and *miRNA-a2/a3/a5/a7* lines. Asterisks indicate significant differences (Student’s t-test, P< 0.05, n=3). Error bars represent the SE. **(F)** Relative expression of *DREB1A* and **(G)** *DREB2A* infour-week-old Wt and *miRNA-a2/a3/a5/a7* lines grown under watered conditions, normalized to the expression of *actin*. Asterisks indicate significant differences (Student’s t-test; P < 0.05 of three biological replicates). Error bar is SE.

**
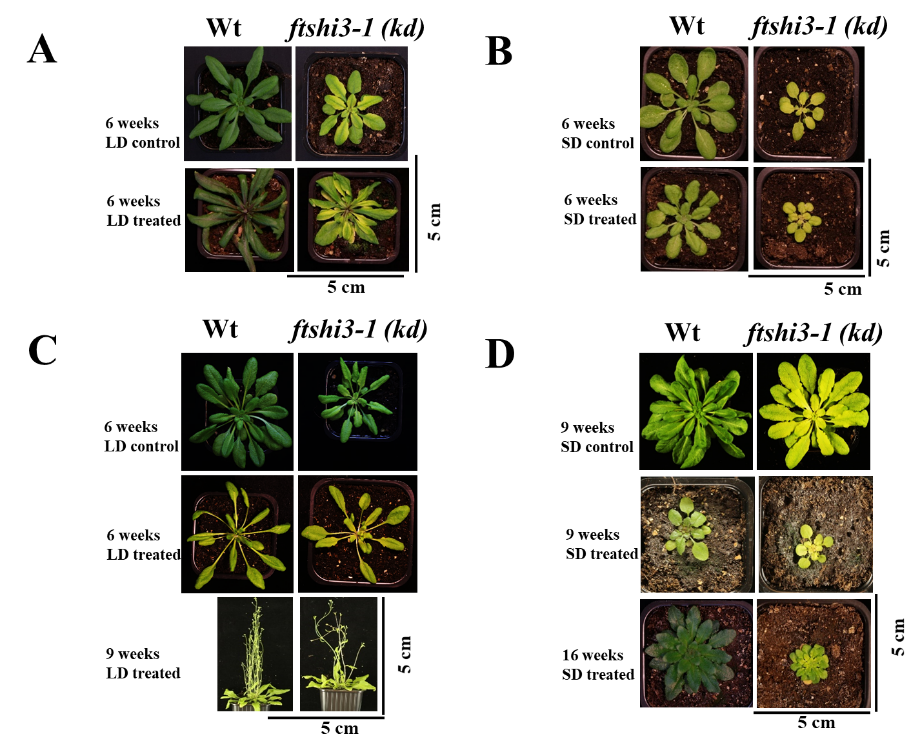
**

**
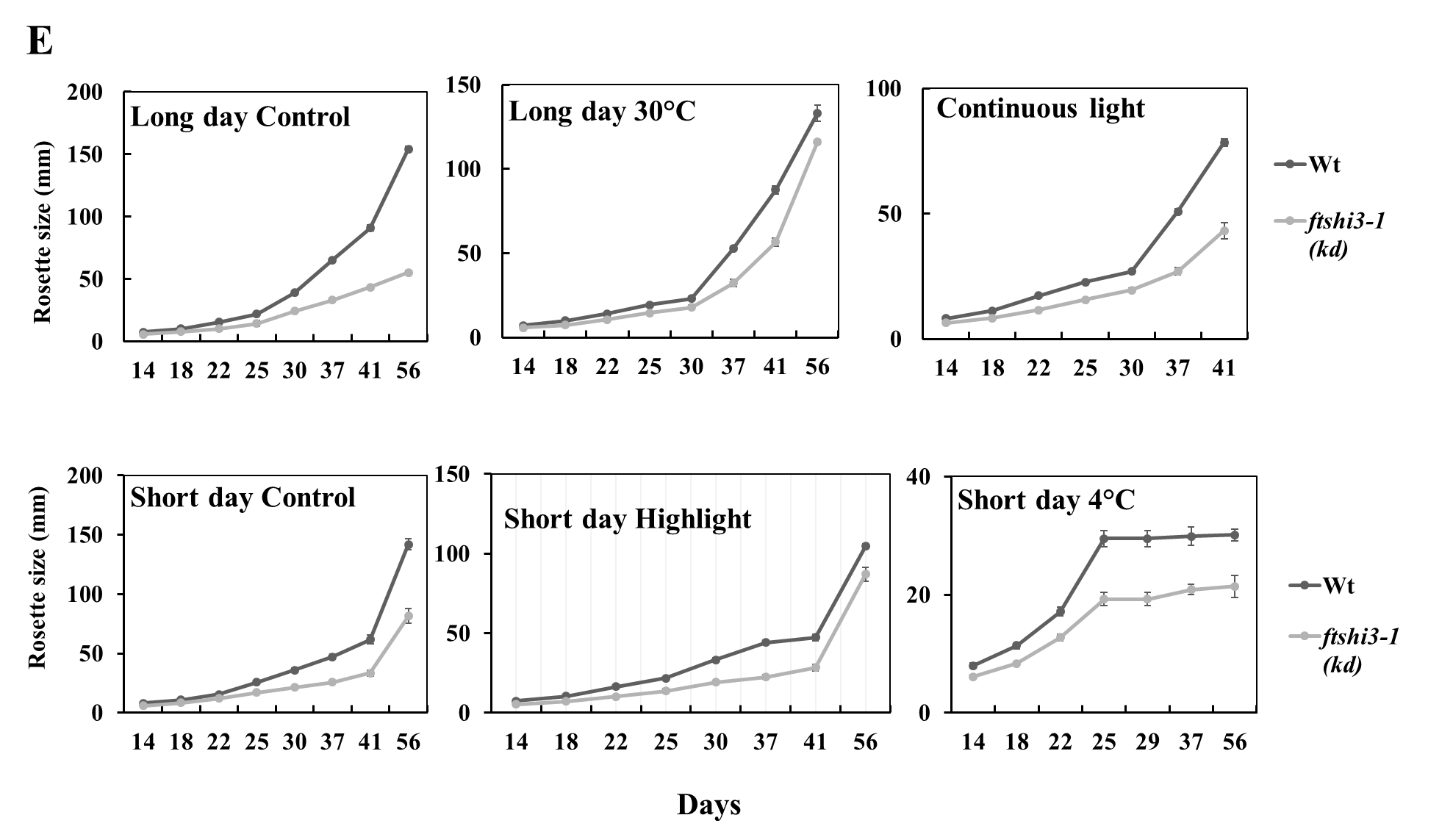
**

**
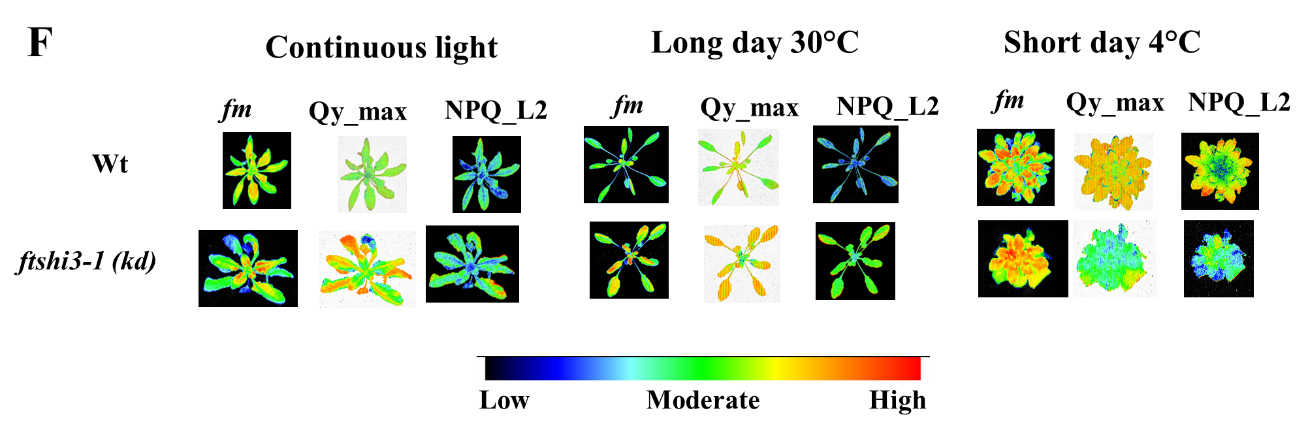
**

**Figure S4**: Phenotypes of Wt and *ftshi3-1(kd)* exposed to stress from the age of 2 weeks to 6, 9 or 16 weeks. **(A)** Exposure to continuous light (150 µmol photons m^-2^ s^-1^); **(B)** high light (700 µmol photons m^-2^ s^-1^) at short day (SD); (C) high temperature (30°C) at long day (LD); **(D)** cold temperature (4°C) at SD. **(E)** Rosette diameter measurements were performed from week 2 to 6 on plants grown at LD (control), 30°C and LD or continuous light (upper panel) as well as on plants grown at SD (control), highlight and SD or low temperature (4°C) and SD (lower panel). **(F)** Representative chlorophyll fluorescence images of the maximum amount of fluorescence (*fm)*, PSII quantum yield (Qy_max) and NPQ during light adaptation (NPQ_L2) for Wt and *ftshi3-1(kd)* after exposure to continuous light (150-µmol photons m^-2^ s^-1^), high temperature (30°C) at LD and cold temperature (4°C) at SD. The colour code depicted below ranges from black (minimum value) to red (maximum value).


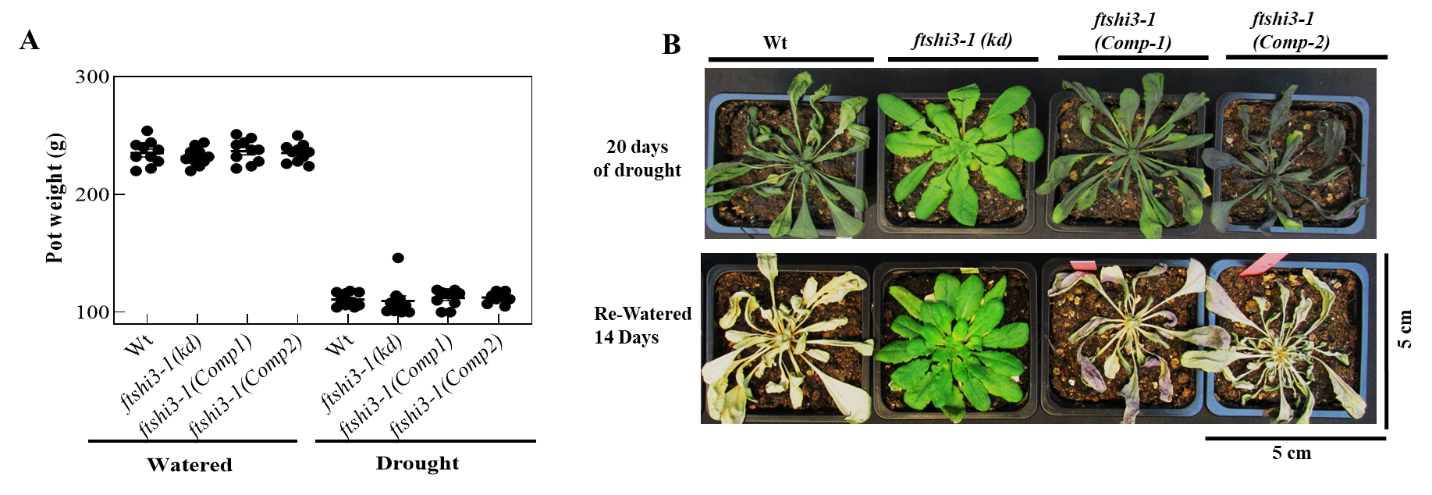



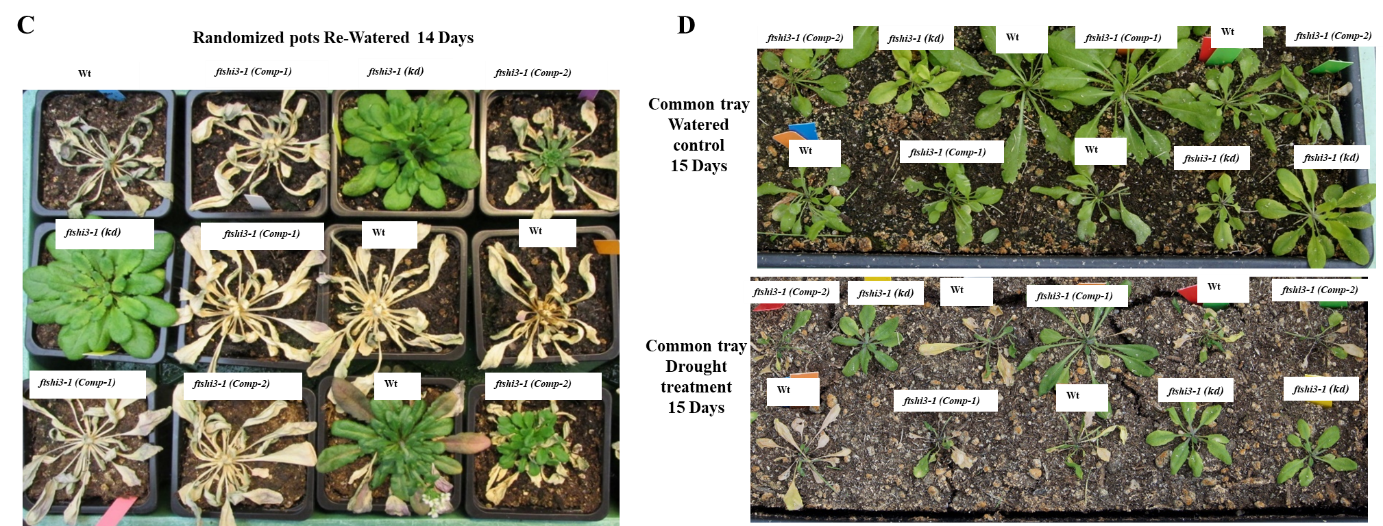


**Figure S5: (A)** Pot weight (g) of Wt*, ftshi3-1(kd)* and *ftshi3-1 (Comp-1* and *Comp-2)* lines in watered and drought conditions (12 days). **(B)** Wt*, ftshi3-1(kd)* and *ftshi3-1 (Comp-1* and *Comp-2)* lines were exposed to drought stress for 20 days (upper panel) and then re-watered for two weeks to determine the rate of recovery (lower panel). **(C)** Randomised pots of Wt*, ftshi3-1(kd)* and *ftshi3-1 (Comp-1* and *Comp-2)* lines within each tray were re-watered for two weeks after exposure to drought stress for 20 days. **(D)** Common tray experiment where Wt*, ftshi3-1(kd)* and *ftshi3-1 (Comp-1* and *Comp-2)* lines were grown in watered (upper panel) and drought (lower panel) conditions (15 days).


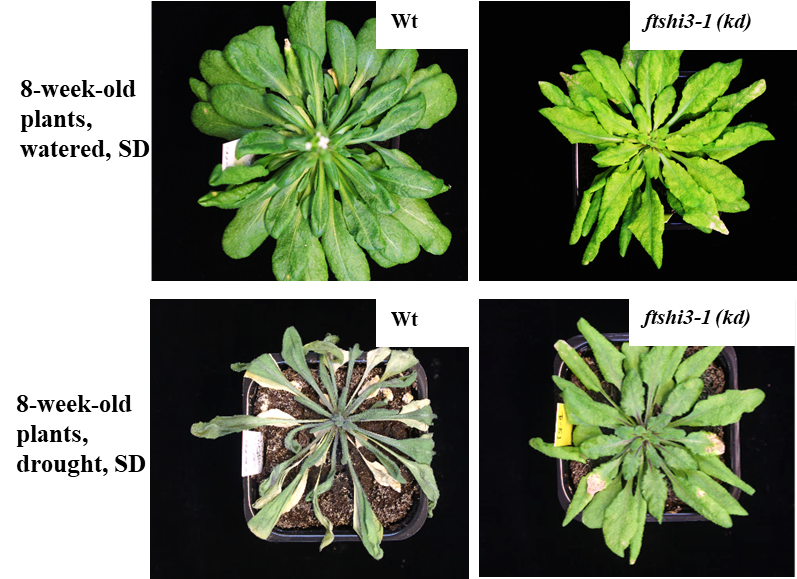


**Figure S6:** Eight-week-old Wt and *ftshi3-1(kd)* plants exposed to watered (control, upper panel) or drought treatment (lower panel) at SD growth conditions.


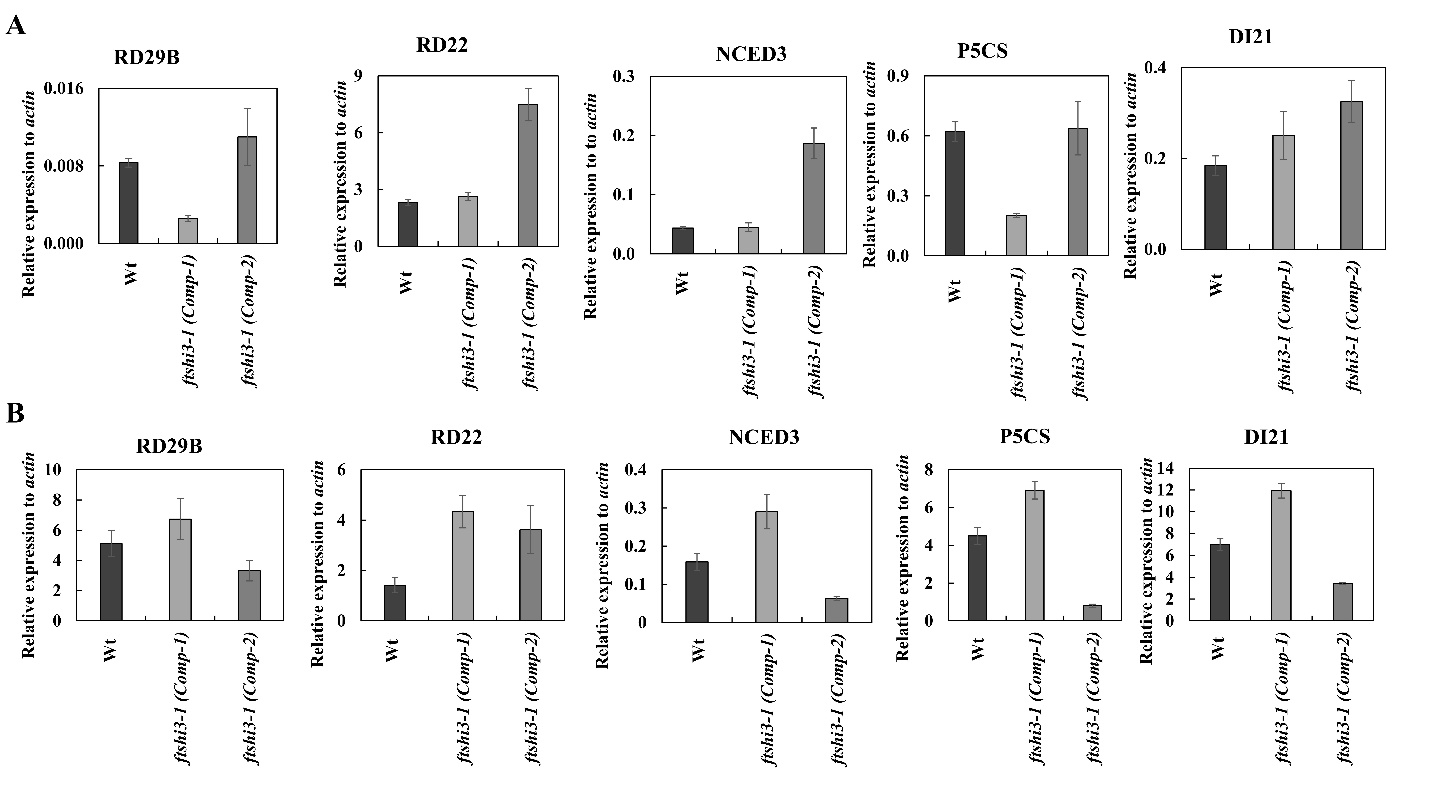


**Figure S7: (A)** Relative expression of ABA-responsive genes at watered conditions in Wt and *ftshi3-1 (Comp-1* and *Comp-2)* lines (three biological replicates each). The data were normalised to the expression of genes coding for *Arabidopsis* actin. **(B)** Relative expression of drought-responsive genes under drought conditions for 12 days in Wt control and *ftshi3-1 (Comp-1* and *Comp-2)* lines (three biological replicates each) normalised to actin expressio*n*. Error bar is SE.


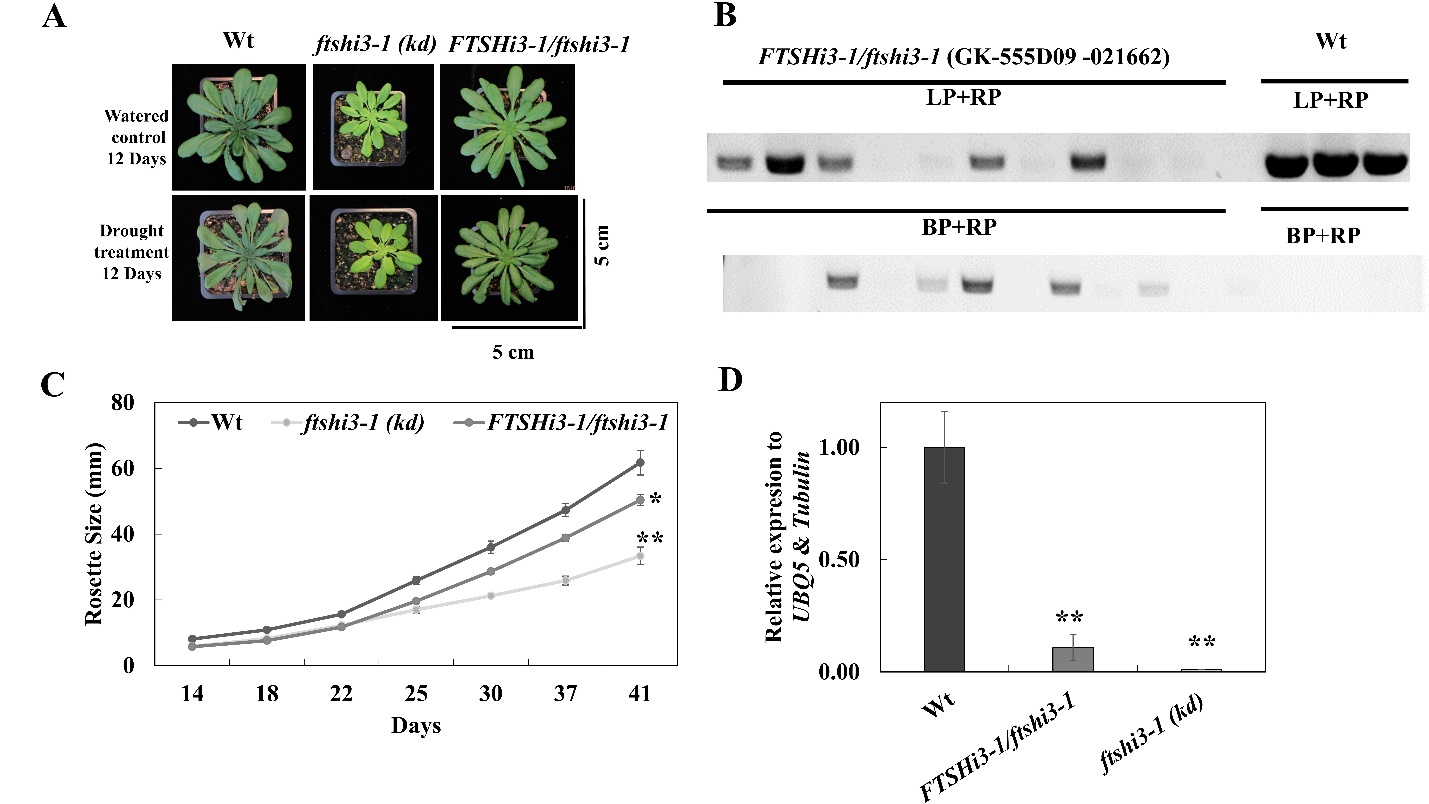


**Figure S8:** (A) Phenotype of Wt*, ftshi3-1(kd)* and *FTSHi3-1*/*ftshi3-1* exposed to drought stress. Eight replicates of four-weeks-old plants of each line were subjected to drought stress by withholding watering for 12 days (lower panel), while the controls were watered (upper panel). (B) Genotyping PCR-gel of *FTSHi3-1*/*ftshi3-1/ftshi3-1* *(GK-555D09-021662).* (C) Rosette diameter measurements were performed from week 2 to 6 on plants grown in SD. Asterisks indicate a significant difference (P < 0.05, Student’s t-test, eight biological replicates represented). (D) Gene expression analysis of Wt, *ftshi3-1(kd)* and *Ftshi3-1*/*ftshi3-1/ftshi3-1* lines. Relative expression was normalised to *At-UBQ5* and *At-Tubulin*. Asterisks indicate a significant difference (P < 0.05, Student’s t-test, three biological replicates represented). Error bar is SE.

**Table S1:** Primers used for genotyping, cloning and qPCR.

| No. | Primer name | Primer sequence | Purpose |
| --- | --- | --- | --- |
| 1 | o8409 | 5’-ATATTGACCATCATACTCATTGC-3’ | Genotyping |
| 2 | o8474 | 5’-ATAATAACGCTGCGGACATCTACATTTT-3’ | Genotyping |
| 3 | GT_i3-1F | 5’-TAGTTGCAGGAGACTTGGTGG-3’ | Genotyping |
| 4 | GT_i3-1R | 5’-GGAAGGTGAGTTTTTCCTTGC-3’ | Genotyping |
| 5 | FLAG_215F10_LP | 5’-CTGTTTCAAAGGTTTTGCTCG-3’ | Genotyping |
| 6 | FLAG_215F10_RP | 5’-CTTCTGGTCGGTTAGTTGCTG-3’ | Genotyping |
| 7 | HSO702_RP | 5’-TACGCATCTGTAAGCTGAGAAA-3’ | Genotyping |
| 8 | HSO702_FP | 5’-AACCCGCCATTTATTCAGTCTA-3’ | Genotyping |
| 9 | hydroxyproline_FP | 5’-AGGTAAAGAAGGGGTGATGCA-3’ | Genotyping |
| 10 | hydroxyproline_RP | 5’-TGACCTCTTCTTGCACCAGA-3’ | Genotyping |
| 11 | AtFtsHi3 promoter_Topo_F | 5’-CACCACTAACCTGAAGAGACTC-3’ | Cloning |
| 12 | AtFtsHi3_withstop_R | 5’-CTAGCTGAGAGTTTGATAACCTAAC-3’ | Cloning |
| 13 | 533_I miR-s | GATGTAATCTAGGTCGACGCCTATCTCTCTTTTGTATTCC-3’ | miRNA cloning |
| 14 | 533_II miR-a | 5’-GATAGGCGTCGACCTAGATTACATCAAAGAGAATCAATGA-3’ | miRNA cloning |
| 15 | 533_III miR*s | 5’-GATAAGCGTCGACCTTGATTACTTCACAGGTCGTGATATG-3’ | miRNA cloning |
| 16 | 533_IV miR*a | 5’-GAAGTAATCAAGGTCGACGCTTATCTACATATATATTCCT-3’ | miRNA cloning |
| 17 | 1952_I miR-s | 5’-GATGTTCATTTCCTAACGTCCTATCTCTCTTTTGTATTCC-3’ | miRNA cloning |
| 18 | 1952_II miR-a | 5’-GATAGGACGTTAGGAAATGAACATCAAAGAGAATCAATGA-3’ | miRNA cloning |
| 19 | 1952_III miR*s | 5’-GATAAGACGTTAGGATATGAACTTCACAGGTCGTGATATG-3’ | miRNA cloning |
| 20 | 1952_IV miR*a | 5’-GAAGTTCATATCCTAACGTCTTATCTACATATATATTCCT-3’ | miRNA cloning |
| 21 | A | 5’-caccCTGAAGGCGATTAAGTTGGGTAAC-3’ | miRNA cloning |
| 22 | B | 5’-GCG GAT AAC AAT TTC ACA CAG GAA ACA G-3’ | miRNA cloning |
| 23 | qi-3-RT-PCR FP | 5’-CCAGACGTTAAACCAGTTGC-3’ | qPCR |
| 24 | qi-3-RT-PCR RP | 5’-CTTCTGGTCGGTTAGTTGC-3’ | qPCR |
| 25 | ACT2-RT-PCR FP | 5’-CTTGCACCAAGCAGCATGAA-3’ | qPCR |
| 26 | ACT2 -RT-PCR FP | 5’-CCGATCCAGACACTGTACTTCCTT-3’ | qPCR |
| 27 | UBQ5-RT-PCR FP | 5’-ACGCTTCATCTCGTC-3’ | qPCR |
| 28 | UBQ5-RT-PCR FP | 5’-CCACAGGTTGCGTTA-3’ | qPCR |
| 29 | Tubulin-RT-PCR FP | 5’-GGTATCCAACCCGATGGCA-3’ | qPCR |
| 30 | Tubulin-RT-PCR FP | 5’-TGAGCTTGTCTCGCTAAAGAATG-3’ | qPCR |
| 31 | DI21-RT-PCR FP | 5’-TCC CTT ACT CAA TCC TGC TGC-3’ | qPCR |
| 32 | DI21-RT-PCR RP | 5’-ACT CTC CGG TGC CGT TAA ATC-3’ | qPCR |
| 33 | DREB1A-RT-PCR FP | 5’-GCG CTA AGG ACA TCC AAA AGG-3’ | qPCR |
| 34 | DREB1A-RT-PCR RP | 5’-GTA AAT AGC CTC CAC CAA CGT C-3’ | qPCR |
| 35 | NECD3-RT-PCR FP | 5’-TTC ATC TGC GCT TCA CAC TCC-3’ | qPCR |
| 36 | NECD3-RT-PCR RP | 5’-GCC GCT CTC TGG AAC AAA TTC-3’ | qPCR |
| 37 | COR47-RT-PCR FP | 5’-GTT GGT TGT AAC GGA GCA TCC-3’ | qPCR |
| 38 | COR47-RT-PCR RP | 5’-CCAAAATCCCCTTCTTCTCCT C | qPCR |
| 39 | P5CS RT-PCR FP | 5’-AGCAGCCTGTAATGCGATGG-3’ | qPCR |
| 40 | P5CS RT-PCR RP | 5’-AAGTGACGCCTTTGGTTTGC-3’ | qPCR |
| 41 | RD22 RT-PCR FP | 5’-AGGGCTGTTTCCACTGAGG-3’ | qPCR |
| 42 | RD22 RT-PCR RP | 5’-CACCACAGATTTATCGTCAGACA-3’ | qPCR |
| 43 | RD29A RT-PCR FP | 5’-GTTACTGATCCCACCAAAGAAGA-3’ | qPCR |
| 44 | RD29A RT-PCR RP | 5’-GGAGACTCATCAGTCACTTCCA-3’ | qPCR |
| 45 | DREB2A RT-PCR FP | 5’-GCAGTTTATGATCAGAG-3’ | qPCR |
| 46 | DREB2A RT-PCR FP | 5’-AACTTCTTCTACGGTCTCGT-3’ | qPCR |
| 47 | RD29B RT-PCR FP | 5´-GGAGTGAAGGAGACGCAACA-3´ | qPCR |
| 48 | RD29B RT-PCR RP | 5´-CCACCTCCTTTGTAGCCGTT-3´ | qPCR |

**Table S2:** Chlorophyll content and chlorophyll *a/b* ratios of rosette leaves of Wt and *ftshi3-1 (kd)* at the age of six weeks. Values are given in µg chlorophyll per mg fresh weight. Asterisks indicate a significant difference (P< 0.05, Student’s t-test, three biological replicates represented). Error bar is SE.

|  | **Chl *a+b* (µg/mg FW)** | **Chl *a/b*** |
| --- | --- | --- |
| **Wt** | **0.94±0.03** | **3.04±0.02** |
| ***ftshi3-1 (kd)*** | **0.49*±0.06** | **2.93*±0.03** |

**Table S3**: Room temperature chlorophyll fluorescence during steady-state conditions of Wt and *ftshi3-1(kd)* exposed to various stress conditions. Measurements were performed on plants at the age of 2 weeks (t=0) and after stress exposure for 3 days, 9 weeks and 16 weeks (cold stress only). Highlight (HL): 700 µmol photons m^-2^ s^-1^, continuous light (CL): 150 µmol photons m^-2^ s^-1^. Values written in red indicate a p-value of less than 0.05, Student’s *t*-test was performed to determine the statistical significance.

**Table. S4**. Pairwise PERMANOVA of Bray-Curtis distances.

| **Group 1** | **Group 2** | **R2** | **P-value** | **Q-value** |
| --- | --- | --- | --- | --- |
| Watered Wt | Watered *ftshi3-1(kd)* | 0.168 | 0.051 | 0.062 |
| Watered Wt | Watered *ftshi3-1(Comp-1)* | 0.124 | 0.245 | 0.274 |
| Watered Wt | Watered *ftshi3-1(Comp-2)* | 0.184 | 0.026 | 0.035 |
| Watered *ftshi3-1(kd)* | Watered *ftshi3-1(Comp-1)* | 0.115 | 0.386 | 0.416 |
| Watered *ftshi3-1(kd)* | Watered *ftshi3-1(Comp-2)* | 0.116 | 0.413 | 0.428 |
| Watered *ftshi3-1(Comp-1)* | Watered *ftshi3-1(Comp-2)* | 0.143 | 0.147 | 0.172 |
| Watered Wt | Drought Wt | 0.233 | 0.008 | 0.045 |
| Watered ftshi3-1*(kd)* | Drought *ftshi3-1(kd)* | 0.273 | 0.015 | 0.023 |
| Watered *ftshi3-1 (Comp-1)* | Drought *ftshi3-1(Comp-1)* | 0.290 | 0.013 | 0.024 |
| Watered *ftshi3-1 (Comp-2)* | Drought *ftshi3-1(Comp-2)* | 0.237 | 0.005 | 0.07 |
| Watered Wt | Drought *ftshi3-1(kd)* | 0.294 | 0.012 | 0.028 |
| Watered Wt | Drought *ftshi3-1(Comp-1)* | 0.258 | 0.012 | 0.028 |
| Watered Wt | Drought *ftshi3-1(Comp-2)* | 0.248 | 0.014 | 0.023 |
| Watered *ftshi3-1(kd)* | Drought Wt | 0.242 | 0.01 | 0.031 |
| Watered *ftshi3-1(kd)* | Drought *ftshi3-1(Comp-1)* | 0.274 | 0.004 | 0.112 |
| Watered *ftshi3-1(kd)* | Drought *ftshi3-1(Comp-2)* | 0.259 | 0.013 | 0.024 |
| Watered *ftshi3-1 (Comp-1)* | Drought Wt | 0.234 | 0.009 | 0.039 |
| Watered *ftshi3-1 (Comp-1)* | Drought *ftshi3-1(kd)* | 0.303 | 0.013 | 0.024 |
| Watered *ftshi3-1 (Comp-1)* | Drought *ftshi3-1(Comp-2)* | 0.257 | 0.01 | 0.031 |
| Watered *ftshi3-1 (Comp-2)* | Drought Wt | 0.215 | 0.009 | 0.039 |
| Watered *ftshi3-1 (Comp-2)* | Drought *ftshi3-1(kd)* | 0.305 | 0.007 | 0.056 |
| Watered *ftshi3-1 (Comp-2)* | Drought *ftshi3-1(Comp-1)* | 0.280 | 0.007 | 0.056 |
| Drought Wt | Drought *ftshi3-1(kd)* | 0.201 | 0.012 | 0.028 |
| Drought Wt | Drought *ftshi3-1(Comp-1)* | 0.178 | 0.016 | 0.024 |
| Drought Wt | Drought *ftshi3-1(Comp-2)* | 0.091 | 0.791 | 0.791 |
| Drought *ftshi3-1(kd)* | Drought *ftshi3-1(Comp-1)* | 0.172 | 0.047 | 0.06 |
| Drought *ftshi3-1(kd)* | Drought *ftshi3-1(Comp-2)* | 0.230 | 0.01 | 0.031 |
| Drought *ftshi3-1 (Comp-1)* | Drought *ftshi3-1(Comp-2)* | 0.187 | 0.023 | 0.032 |

**Table S5:** Summary of the indicator species analyses, listing genera that are associated with different watering treatments and/or host genotypes.

*Excel sheet attached.*
